# From microstructures to macroclimates: SEM-based differentiation and MaxEnt habitat suitability of *Hypocassida subferruginea* and *Cassida palaestina* in Türkiye

**DOI:** 10.1515/biol-2025-1384

**Published:** 2026-10-02

**Authors:** Neslihan Bal, Damla Amutkan Mutlu, Mesut Sirri

**Affiliations:** Department of Biology, Gazi University, Science Faculty, 06500 Ankara, Türkiye; Department of Crop and Animal Production, Kurtalan Vocational School, Siirt University, Siirt, Türkiye

**Keywords:** Cassidini, SEM, *Hypocassida*, MaxEnt, habitat suitability

## Abstract

This study integrates scanning electron microscopy (SEM) and MaxEnt ecological niche modeling to investigate morphological differentiation and climate-driven habitat suitability of two Cassidini species in Türkiye, *Hypocassida subferruginea* (Schrank, 1776) and *Cassida palaestina* Reiche & Saulcy, 1858. SEM analyses revealed distinct diagnostic differences, including the deep longitudinal antennal grooves beneath the pronotum in *Hypocassida*, supporting genus-level divergence within Cassidinae. Additional differences were observed in antennal sensilla, sternal structures, and tarsomere morphology. The pupal stage of *H. subferruginea* is described for the first time, while the fifth instar larva of *C. palaestina* is documented using SEM. Immature stages provided previously underreported characters, including cauliflower-shaped sensilla, scoli differentiation, and spiracular-associated structures, contributing new diagnostic traits for Cassidini morphology. Habitat suitability was modeled using occurrence records and WorldClim v2.1 bioclimatic variables. Models showed high predictive performance (AUC = 0.942 for *H. subferruginea*; 0.997 for *C. palaestina*). Temperature variables were the primary drivers of *H. subferruginea* distribution, whereas both temperature and precipitation influenced *C. palaestina*. Predicted distributions indicated a clear north–south ecological gradient in Türkiye. The combined use of SEM and ecological modeling provides valuable insights into taxonomy, species differentiation, and ecological divergence within Cassidini.

## 1 Introduction

The tribe Cassidini within the subfamily Cassidinae Gyllenhal, 1813 comprises a total of 15 genera: *Cassida* Linnaeus, 1758; *Chiridopsis* Spaeth, 1922; *Chiridula* Weise, 1889; *Glyphocassis* Spaeth, 1914; *Hypocassida* Weise, 1893; *Ischyronota* Weise, 1891; *Macromonycha* Spaeth, 1911; *Nabathaea* Spaeth, 1911; *Oocassida* Weise, 1897; *Oxylepus* Desbrochers des Loges, 1884; *Pilemostoma* Desbrochers des Loges, 1891; *Rhytidocassis* Spaeth, 1941; *Seminabathea* Borowiec, 1994; *Thlaspida* Weise, 1899; and *Thlaspidosoma* Spaeth, 1901 [1].

Among these 15 genera, the most significant ones found in Turkey are *Hypocassida* Weise, 1893 and *Cassida* Linnaeus, 1758.

Of the five species of the genus *Hypocassida* known from the Palaearctic region, three species are present in Turkey [1].

– *Hypocassida cornea* Marseul, 1868
– *Hypocassida meridionalis* Suffrian, 1844
– *Hypocassida subferruginea* Schrank, 1776

The genus *Cassida* Linnaeus, 1758 is represented by 170 species in the Palaearctic region, 41 of which are found in Turkey. These include [1]:

– *Cassida algirica* Boheman, 1847;
– *Cassida alidagiensis* Özdikmen, Coral Şahin & Bal, 2020;
– *Cassida atrata* Fabricius, 1787;
– *Cassida azurea* Fabricius, 1801;
– *Cassida bella* Faldermann, 1837;
– *Cassida berolinensis* Suffrian, 1844;
– *Cassida brevis* Weise, 1884;
– *Cassida canaliculata* Laicharting, 1781;
– *Cassida denticollis* Suffrian, 1844;
– *Cassida elongata* Weise, 1893;
– *Cassida fausti* Spaeth, 1926;
– *Cassida ferruginea* Goeze, 1777;
– *Cassida flaveola* Thunberg, 1794;
– *Cassida hablitziae* Motschulsky, 1838;
– *Cassida hemisphaerica* Herbst, 1799;
– *Cassida inquinata* Brullé, 1833;
– *Cassida lineola* Creutzer, 1799;
– *Cassida linnavuorii* Borowiec, 1986;
– *Cassida margaritacea* Schaller, 1783;
– *Cassida murraea murraea* Linnaeus, 1767;
– *Cassida nebulosa* Linnaeus, 1758;
– *Cassida nobilis* Linnaeus, 1758;
– *Cassida palaestina* Reiche & Saulcy, 1858;
– *Cassida pannonica* Weise, 1892;
– *Cassida parvula* Boheman, 1854;
– *Cassida prasina* Illiger, 1798;
– *Cassida pusilla* Waltl, 1835;
– *Cassida rubiginosa rubiginosa* O. F. Müller, 1776;
– *Cassida rufovirens* Suffrian, 1844;
– *Cassida sanguinolenta* O. F. Müller, 1776;
– *Cassida sanguinosa* Suffrian, 1844;
– *Cassida saucia* Weise, 1889;
– *Cassida seladonia* Gyllenhal, 1827;
– *Cassida seraphina* Ménétriés, 1836;
– *Cassida stigmatica* Suffrian, 1844;
– *Cassida strejceki* Sekerka, 2006;
– *Cassida subreticulata* Suffrian, 1844;
– *Cassida transcaucasica* Borowiec & Świętojańska, 2001;
– *Cassida vibex* Linnaeus, 1767;
– *Cassida viridis* Linnaeus, 1758;
– *Cassida vittata* Villers, 1789. One of these species is *Cassida palaestina.*

The Cassidinae subfamily exhibits remarkable morphological, ecological, and behavioral diversity. The size of adult individuals varies widely, ranging from the very small representatives of the genus *Oxylepus* (approximately 3 mm in length) to the relatively large members of the genus *Alurnus* (up to 30 mm in length). Larvae show a similar size range, from 1 mm to 40 mm; in particular, *Alurnus* larvae can reach lengths of up to 40 mm [2]. The term *Cassida*, along with the common name “tortoise beetles,” is used to describe species characterized by a rounded body shape marked by the explanate (flared) pronotum and elytra as well as the retracted position of the head beneath the pronotum [3].

The subfamily Cassidinae exhibits a wide range of host plant preferences, encompassing species that are polyphagous (feeding on numerous plant species), narrowly oligophagous (restricted to specific plant genera), or monophagous (specialized on a single plant species) [4]. Their ability to feed on a variety of cultivated plants makes members of the family Chrysomelidae one of the most significant groups of agricultural pests. While foliar feeding causes varying degrees of damage to host plants, high population densities and periodic outbreaks can render both larvae and adults of Cassidinae serious pests [5], 6]. These insects often live by boring into plant stems or hiding cryptically within rolled-leaf tubes or floral bracts [7]. In addition to their role as phytophagous pests, Cassidinae species may also have economically beneficial aspects. Notably, some species serve as biological control agents against certain weedy plants, thereby contributing positively to integrated pest management strategies [8].

One of the most remarkable behaviors observed in tortoise beetles is the recycling of feces and exuviae (shed cuticle remnants) to construct a defensive shield. This shield is carried on the dorsum by the larvae and is sometimes retained even during the pupal stage. Known as “fecal recycling,” this behavior is not exclusive to the Cassidinae subfamily; similar strategies such as the coating of eggs with feces or the lining of pupal chambers with fecal material have also been documented in various other Chrysomelidae Species [9]. The shield may be composed entirely of exuviae or a combination of feces and exuviae [10], 11]. Shield-bearing larvae exhibit morphological adaptations related to the construction, maintenance, and carriage of this structure. A key adaptation is the presence of a flexible, telescopic anus, formed by abdominal segment *X* and an elongation of the anus, which allows the larvae to repair or rebuild the shield in case of damage [4].

A major challenge in the systematics of *Cassida*, and indeed throughout the *Cassidinae*, is the limited understanding of species’ life histories and ecological traits, along with the scarcity of specimens representing all life stages for comprehensive comparative studies. Such information would provide novel characters and character states, which are crucial for identifying more natural, monophyletic groups and for clarifying evolutionary relationships within the subfamily [12]. Larvae of *Cassidinae* have been the focus of increased taxonomic attention in recent years. New descriptions have been added for previously undescribed larvae of known species, contributing to a broader understanding of the group’s morphological and taxonomic diversity [13], [14], [15], [16].

Morphological descriptions of adults and larvae within the subfamily Cassidinae are of critical importance for understanding the taxonomy and evolutionary relationships of this group. Notably, significant contributions to Cassidinae systematics have been made under the leadership of Jolanta Świętojańska. Hawkeswood et al. [17] were the first to provide detailed descriptions of the larval, pupal, and adult stages of *Cassida compuneta* (Boheman), an Australian endemic species, laying the foundation for understanding its life cycle and ecology [17].

Świętojańska [18] conducted detailed examinations and illustrations of the first instar larvae of *Cassida nobilis* and *Cassida vittata* from the Palearctic region. She also elucidated the morphological differences among these species and others, including *Cassida nebulosa*, *Cassida stigmatica*, and *Cassida rubiginosa*. This work highlighted the presence of diagnostic morphological characters related to defensive behaviors in the larvae of closely related species, emphasizing the taxonomic value of larval morphology [18].

Another significant study by Świętojańska [19] provided the first detailed description of the immature stages of *Cassida koreana* Borowiec and Cho [20], and compared them with its close relative *Cassida vespertina*. This comparison, supported by scanning electron microscopy (SEM) images, chaetotaxy patterns, and live photographs, enhanced the depth of morphological analyses [19], 20].

In 2013, Świętojańska and colleagues comprehensively described the larvae of *Cassida palaestina* Reiche [21] and *Cassida rubiginosa* Müller, 1776, accompanied by SEM microstructural data. Based on morphological findings and geographic distribution, it was proposed that these two taxa represent partially parapatric and locally differentiated (vicariant) species. This finding demonstrates that larval morphology, alongside adult morphological characters, provides a reliable basis for species delimitation [21].

More recently, Świętojańska et al. [22] again detailed the immature stages of *Cassida koreana* and compared them with *Cassida vespertina*. The study incorporated SEM imagery and chaetotaxy data, offering valuable evidence for the systematic placement of these Species [22].

Finally, Begha and Oliveira [23] updated the morphological knowledge of *Hybosa acutangula* Spaeth, 1913 by describing larval, pupal, and adult genital structures for the first time. Their research underscores the ongoing need for detailed morphological comparisons to better resolve the taxonomy and evolutionary history within Cassidinae.

In this study, we provide the first comprehensive and systematic comparative analysis of scanning electron microscope (SEM) imagery of adult specimens from two phylogenetically distinct genera within the Cassidini tribe *Hypocassida subferruginea* and *Cassida palaestina* both of which represent some of the most widely distributed and ecologically significant species in Türkiye. This work addresses a critical gap in the literature, as these taxa have not been previously subjected to detailed morphological comparison. Additionally, we offer the inaugural morphological description of the pupal stage of *Hypocassida subferruginea*, accompanied by an in-depth SEM-based examination and documentation of the fifth instar larval morphology of *Cassida palaestina*. These findings not only enhance taxonomic resolution but also contribute valuable insights into the developmental biology and evolutionary relationships within Cassidini [23].

## 2 Materials and methods

*Hypocassida subferruginea* specimens were collected in 2025 by Dr. Mesut SIRRI from Dara village, central Yüksekova, Hakkâri, feeding on *Convolvulus arvensis* L. (Figure 2A–E). Meanwhile, *Cassida palaestina* individuals were gathered from the central Siirt region (between the city center and Kurtalan road), associated with *Notobasis syriaca* (L.) Cass. plants (Figure 1A–D). A total of 12 adult specimens and 3 pupae of *Hypocassida subferruginea*, together with 12 adult specimens and 4 fifth-instar larvae of *Cassida palaestina*, were examined in this study. All specimens were initially examined under stereomicroscopes, and representative, well-preserved individuals were subsequently selected for detailed scanning electron microscopy (SEM) observations (Figure 2).

**Figure 1:**
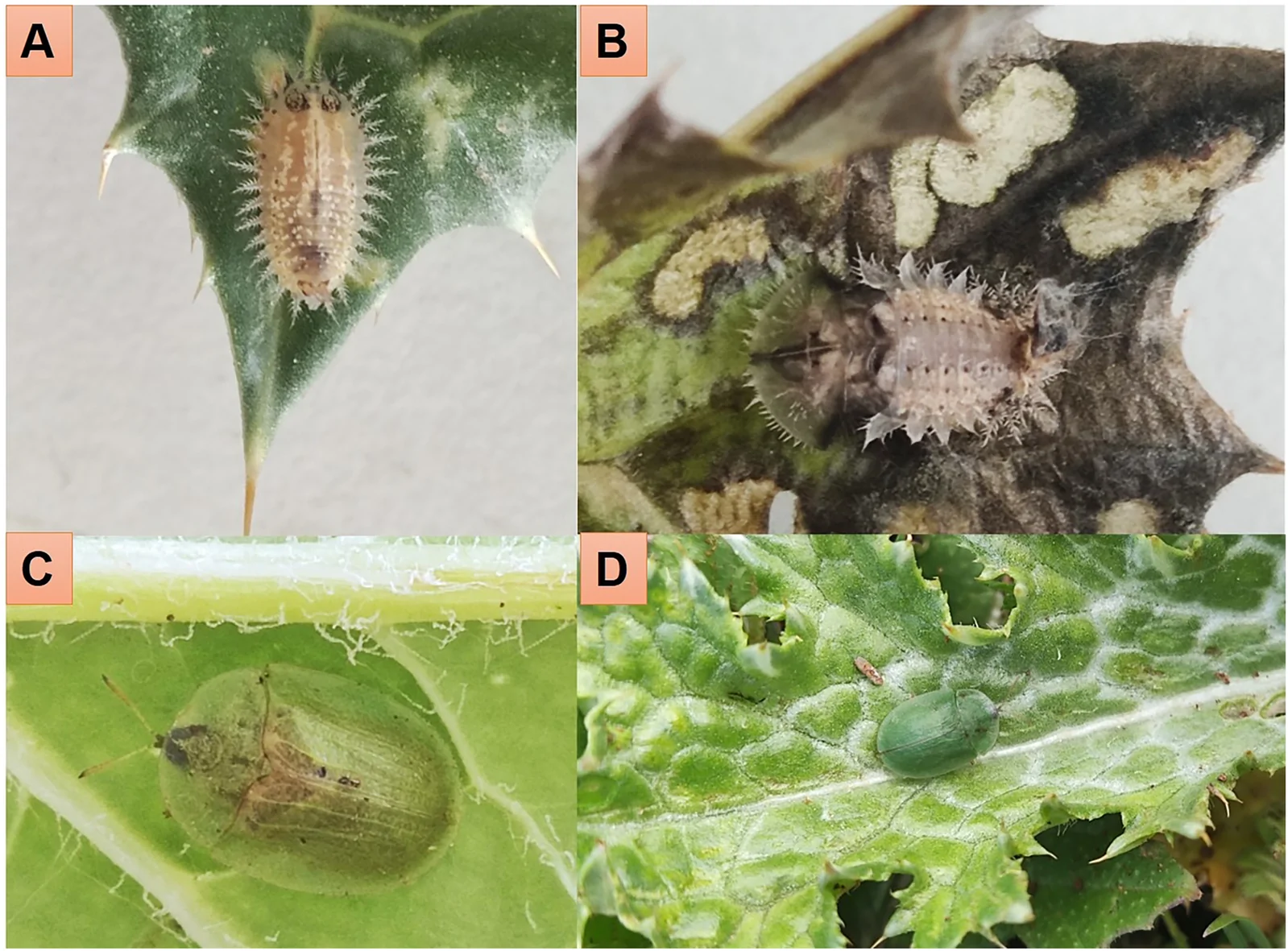
Field observations and developmental stages of *Cassida palaestina*. (A, B) Leaf damage and fifth instar larva of *Cassida palaestina*; (C, D) adult individual of *Cassida palaestina* on *Notobasis* *syriaca*.

**Figure 2:**
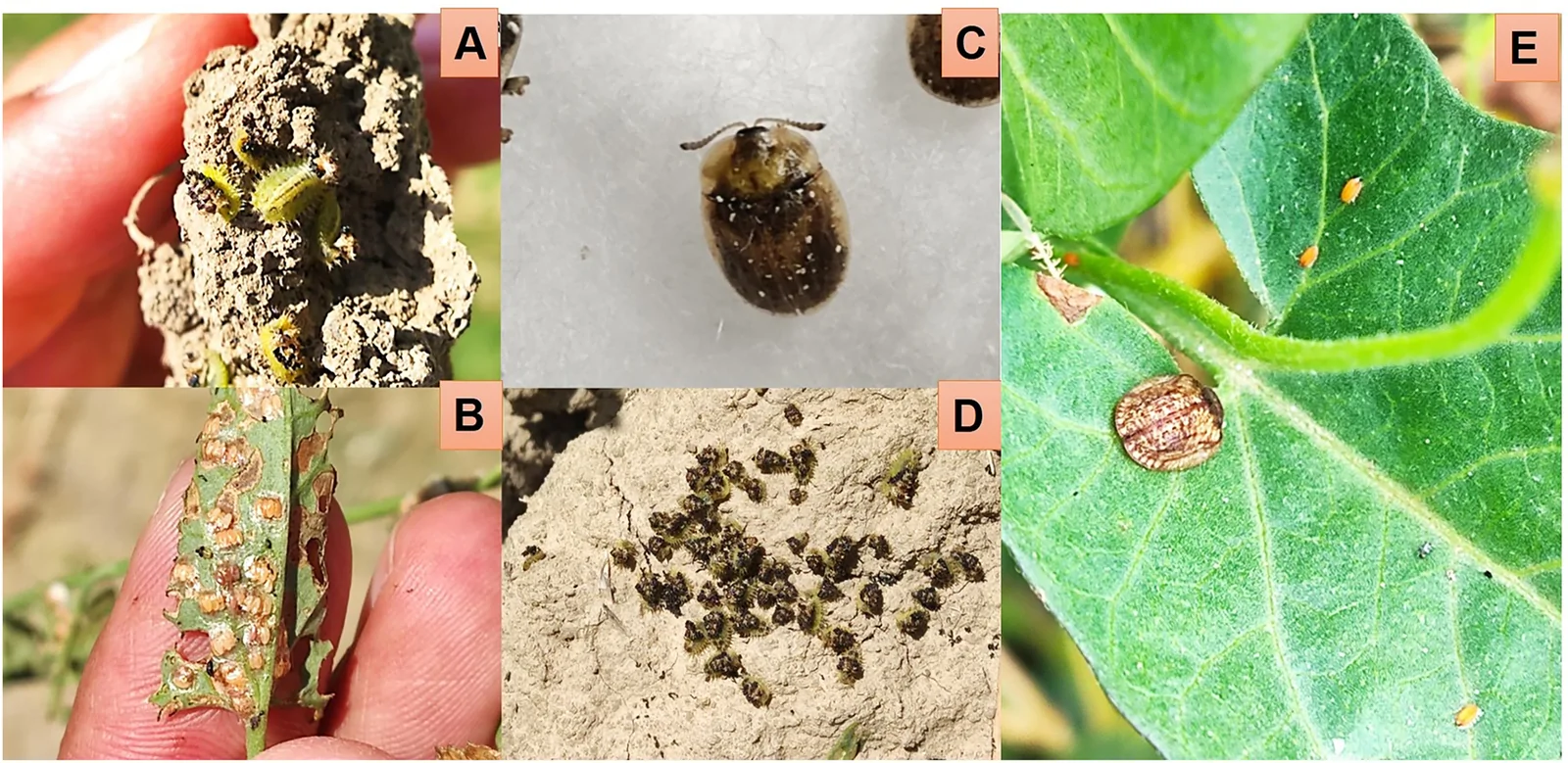
Field observations and developmental stages of *Hypocassida subferruginea*. (A, B) Egg clusters of *Hypocassida subferruginea* and the damage caused to the plant; (C) adult individual of *Hypocassida subferruginea*; (D) fifth instar larva of *Hypocassida subferruginea*; (E) adult individual of *Hypocassida subferruginea* on *Centaurea behen*.

The comparative morphology of *H*. *subferruginea* and *C*. *palaestina*, the pupa of *H*. *subferruginea*, and the detailed morphology of the fifth instar larva of *C*. *palaestina* will be described using scanning electron microscopy (SEM). The specimens were deposited at Gazi University (Ankara, Turkey). Specimen cleaning was performed using 70 % ethanol. Olympus SZX7 and Leica Z-16 APO stereomicroscopes were used during the study.

For scanning electron microscopy (SEM) analysis, specimens fixed in glutaraldehyde were rinsed in a sodium phosphate buffer solution. They were then subjected to a graded ethanol dehydration series at concentrations of 70 %, 80 %, 90 %, and 100 %, with each step lasting 15 min. Following dehydration, the samples were air-dried. Subsequently, the specimens were mounted onto standard aluminum SEM stubs pre-coated with double-sided adhesive tape. The mounted samples were sputter-coated with gold using a Polaron SC 502 Sputter Coater. Finally, imaging and evaluation were carried out at 5–10 kV using a JEOL JSM 6060 SEM housed at the Prof. Dr. Zekiye Suludere Electron Microscopy Center, Gazi University.

SEM micrographs were obtained at magnifications ranging from × 18 to × 2,500, depending on the morphological structures examined. Low magnifications (×18–×70) were used to document the general body morphology and overall structural organization, whereas intermediate magnifications (×70–×500) enabled detailed observation of regions such as the head capsule, thoracic segments, and appendages. Higher magnifications (×500–×2,500) were employed to investigate ultrastructural features, including sensilla types, cuticular surface ornamentation, and fine microstructural details. The scale bars provided in the micrographs ranged from approximately 1 mm at lower magnifications to 10 μm at higher magnifications, allowing accurate interpretation of structural dimensions. All images were acquired at an accelerating voltage of 5–10 kV to optimize surface resolution and minimize charging effects.

Compilation of information on the distribution areas of the target species. Information on the distribution areas of the target species was compiled through a comprehensive literature survey conducted across multiple databases. These included Web of Science, Scopus, and various national and international databases, such as DergiPark, YÖK National Thesis Center (YÖKTEZ), SpringerLink Journals, ScienceDirect, PubMed, and Google Scholar. In addition to the literature review, existing distribution records were downloaded from the Global Biodiversity Information Facility (GBIF, 2024) database [24].

The collected records were checked for taxonomic and georeferencing quality. Records with missing or inaccurate coordinates (coordinate uncertainty> 10 km), duplicates, and questionable geographic locations (e.g., ocean or inland water bodies) were removed before modeling [25].

Cleaned occurrence records were spatially thinned to reduce the effects of spatial autocorrelation and sampling bias stemming from uneven collection effort [26]. Raster-cell thinning was used to retain at most one record per grid cell at the modeling resolution [27]. Spatial thinning was performed in R using the ‘terra’ package for allocating occurrence points to raster cells and ‘dplyr’ package for retaining a single record per cell [28], 29]. This strategy reduced geographical clustering and prevented the repeated contribution of numerous occurrence points within the same environmental grid cell.

### 2.1 Modeling studies

The final dataset obtained from the literature review and the GBIF database was analyzed using the MaxEnt environmental niche modeling approach. This method was selected because it is one of the most widely used tools in biodiversity and ecological distribution studies. In addition, the comparatively strong performance of MaxEnt relative to available alternative modeling approaches was a decisive factor in its selection [30].

Environmental variables and species distribution data used in the modeling were obtained from the WorldClim Version 2.1 dataset. This dataset provides global climate layers at a spatial resolution of 1 km^2^ and consists of 19 bioclimatic variables [31], 32]. To determine the potential habitat suitability of the species in Türkiye, raster datasets were generated; first, the global distribution of the species was modeled, followed by an evaluation of habitat suitability at the national scale, and the results were subsequently mapped. All spatial analyses were conducted using ArcGIS 10.7 software.

During the preliminary modeling stage carried out in the MaxEnt program, the bioclimatic variables that contributed most strongly to the model were identified from the initial set of 19 variables, and only these selected variables were used in subsequent analyses. To eliminate multicollinearity among the bioclimatic variables, Pearson correlation analysis was applied, and variables with a correlation coefficient greater than 0.7 were excluded from the model. As a result, model calibration was performed using seven bioclimatic variables for *H. subferruginea* and six bioclimatic variables for *C. palaestina* (Table 1).

**Table 1:** Relative contribution (%) and permutation importance (%) of the bioclimatic variables included in the MaxEnt model for predicting the habitat suitability of *H. subferruginea* and *C. palaestina* under current climatic conditions.

***H. subferruginea***	**Environmental variables description**	**Bioclimatic**	**Unit**	**Percent contribution**	**Permutation importance**
	Annual mean temperature	bio1	°C	2.2	19.3
	Mean diurnal range (mean of monthly [max temp − min temp])	bio2	°C	8.2	4
	Isothermal temperature (bio2/bio7) ( × 100)	bio3	Index	6	9.7
	Maximum temperature of the hottest month	bio5	°C	3	24.5
	Minimum temperature of the coldest month	bio6	°C	47.2	22.6
	Mean temperature of the hottest Quarter	bio10	°C	1.5	18.2
	Precipitation seasonality (coefficient of variation)	bio15	Index	32	1.7
***C. palaestina***	Mean diurnal range (mean of monthly [max temp − min temp])	bio2	°C	6.7	1.7
	Mean temperature of Wettest Quarter	bio8	°C	30.3	59.9
	Mean temperature of the driest Quarter	bio9	°C	29.9	20.5
	Precipitation of Driest Quarter	bio17	mm	0.0	13.9
	Precipitation of the hottest Quarter	bio18	mm	7	0.0
	Precipitation of the coldest Quarter	bio19	mm	12.2	3.3

### 2.2 Modeling approach

Using MaxEnt version 3.4.4, 75 % of the occurrence records were randomly selected as training data for model calibration, while the remaining 25 % were used as test data for model validation [33]. In addition to the default settings of the MaxEnt software [32], the model was run with 10 replicates, using subsample as the replicated run type, and a maximum of 5,000 iterations [30]. A total of 10,000 background points were randomly generated across the enter study area. During model execution, all predictor variables were treated as continuous variables (e.g., climatic variables). Model was calibrated on a global scale using all 19 climatic parameters initially and only the variables with the highest contribution towatds model training and permutation importance were included in the final model. The global habitat suitability rasters were downscaled to Türkiye, classified, and interpreted. All spatial analyses were performed using ArcGIS 10.7 software. The continuos ratsers were classified as unsuitable (0.0–0.25), moderately suitable (0.26–0.50), suitable (0.51–0.75) and highly suitable (>0.75) [34]. The area corresponding to each class was calculated by raster calculation tool in ArcGIS (ESRI (Environmental Systems Resource Institute), 2012).

## 3 Results and discussion

The most distinctive characteristic of the genus *Hypocassida*, differing from the genus *Cassida*, is the presence of a groove under the pronotum in which the antennae are concealed (Figure 3A–C) (On the underside of the pronotum run two deep longitudinal gutters reaching the hind margin). In *C*. *palaestina*, the pronotum is broad and semicircular, whereas in *Hypocassida* it is narrower and more elliptical; the distance between the uppermost part of the head (ventral view) and the pronotum is quite short in *Hypocassida*, while in *C*. *palaestina* it is much more distinct and pronounced. In addition, the posterior margins of the pronotum in *H*. *subferruginea* terminate more rounded, whereas in *C*. *palaestina* they end more sharply. The clypeus of *H. subferruginea* is longer and narrower, while in *C. palaestina* it is shorter and broader. SEM observations revealed that the clypeus of *H. subferruginea* bears numerous and elongated sensilla, whereas in *C. palaestina* they are sparser and shorter. Moreover, the area between the clypeus and the eyes is covered with a single row of sensilla of almost equal size in *Hypocassida*, while in *C. palaestina* two or more rows of sensilla are evident. The labrum exhibits similar characteristics in both species, being rectangular in shape with a notch in the middle of its lower margin.

**Figure 3:**
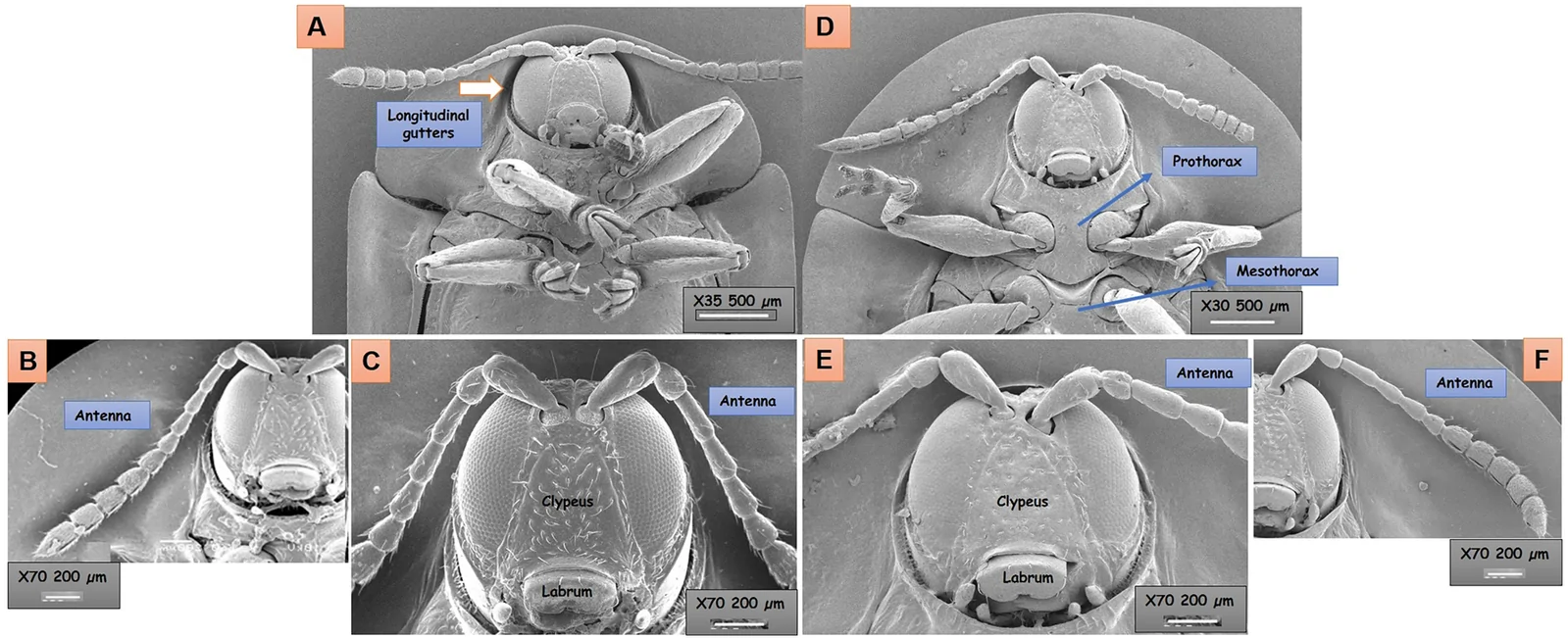
Comparative morphology of the head, antennae, and thoracic regions of *Hypocassida subferruginea* and *Cassida palaestina. *(A) Ventral view of the head, antennae, prothorax, mesothorax, and first two pairs of legs of *Hypocassida subferruginea*; (B, C) antennal and head structures of *H. subferruginea* (labrum, clypeus, eyes); (D) ventral view of the head, antennae, prothorax, mesothorax, and first two pairs of legs of *Cassida palaestina*.

In the examined species representing the two genera, the groove on the last five antennal segments is noteworthy. Toward the terminal antennal segments, both the types and the number of sensilla were observed to increase. In both species, except for the terminal antennal segment, the segments appear almost equal in length and width, giving them a square-like shape, whereas the last segment is conical in form (Figure 4A–D).

**Figure 4:**
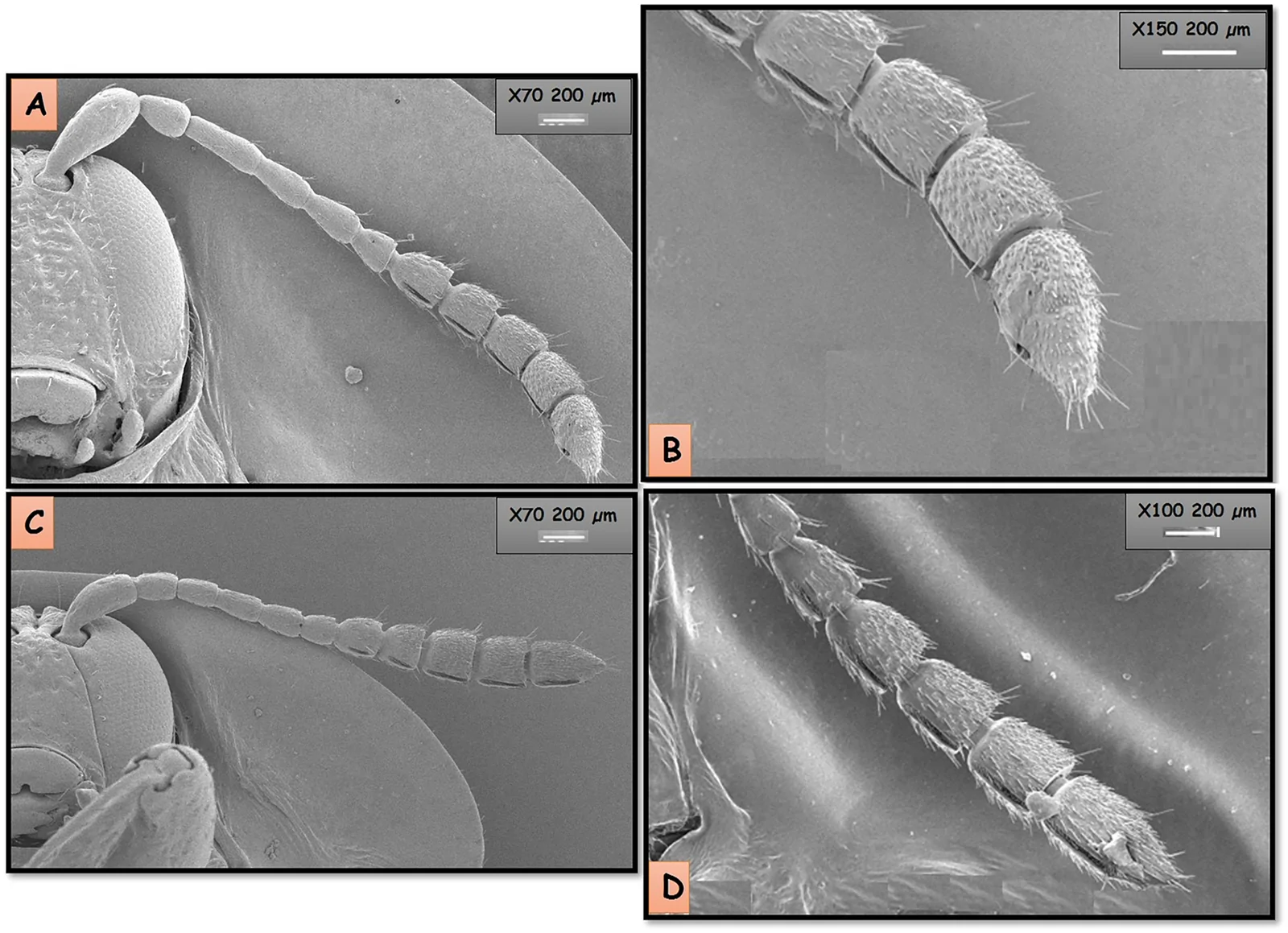
Comparative antennal morphology of *Cassida palaestina* and *Hypocassida subferruginea*. (A) Whole antenna of *Cassida palaestina*; (B) last five antennal segments of *C. palaestina*; (C) whole antenna of *Hypocassida subferruginea*; (D) last five antennal segments of *H. subferruginea*.

Within the *Cassida* group, the proportional ratios of antennal segments represent key diagnostic characters and play an important role in species identification. Comparative examination of two species belonging to different genera revealed clear distinctions in antennal morphology, particularly at the sensilla level (Figure 6A–G). The number, type, and distribution density of sensilla differ markedly between *C*. *palaestina* and *H*. *subferruginea*.

Three types of sensilla trichodea and sensilla chaetica are present in both species; however, in *H*. *subferruginea*, sensilla trichodea are densely distributed on the last four antennal segments, while the number of sensilla chaetica gradually increases toward the apical end (Figure 6H). In contrast, in *C*. *palaestina*, sensilla coeloconica and sensilla ampullacea are additionally observed on the second antennomere (Figure 5A–E).

**Figure 5:**
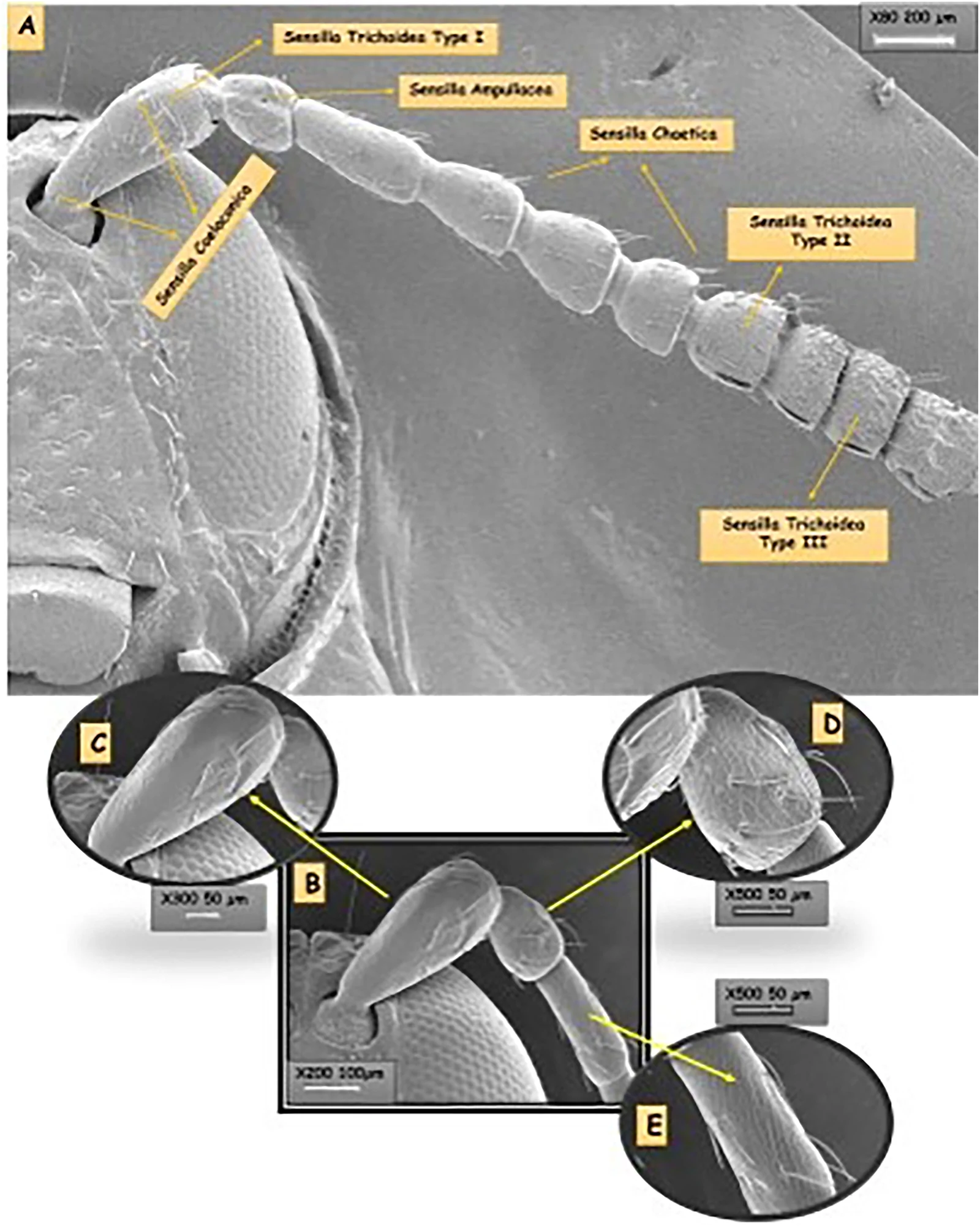
Antennal morphology and sensilla of *Cassida palaestina*. (A) Sensilla structures on the antennal segments of *Cassida palaestina*; (B) first three antennal segments of *Cassida palaestina*; (C) first antennal segment; (D) second antennal segment; E. Third antennal segment.

These morphological variations in sensilla type and arrangement likely reflect differences in sensory function and ecological adaptation between the two genera (Figure 6).

**Figure 6:**
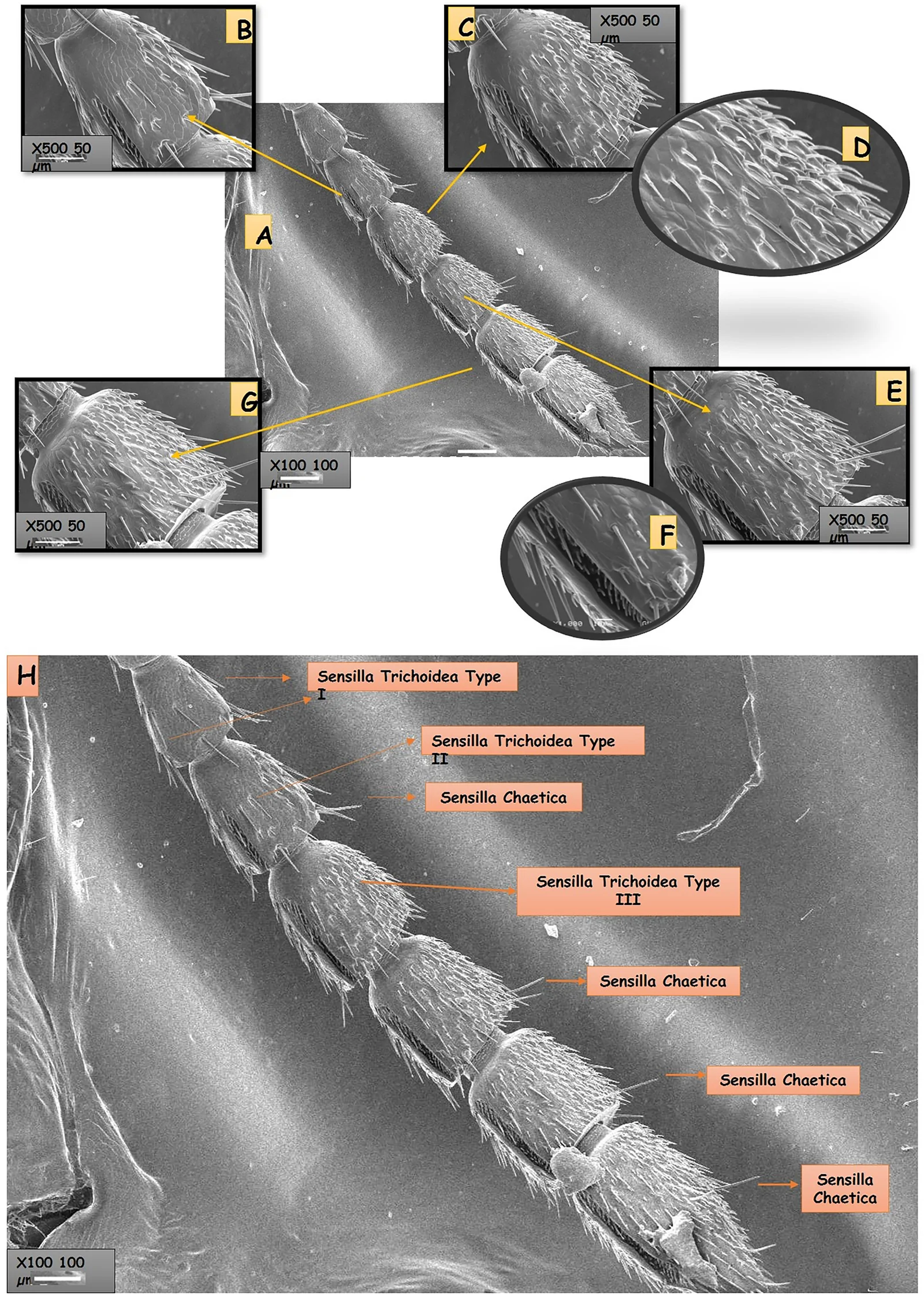
Antennal morphology and sensilla of *Hypocassida subferruginea*. (A) The last six antennal segments of *Hypocassida subferruginea*; (B) the seventh antennal segment; (C) the eighth antennal segment; (D) sensilla on the eighth antennal segment; (E) the ninth antennal segment; (F) sensilla and deep striations on the ninth segment; (G) the tenth antennal segment, (H) sensilla structures on the antennal segments of *Hypocassida subferruginea.*

Sensilla trichodea I is the most common type on the antenna of *Cassida*. It occurs on most antennomeres, but its number varies distinctly from the base to the apex, with a sharp increase in density between the fourth and fifth antennomeres. This sensillum is long, slender, and conical, and is aligned with the longitudinal axis of the antenna. Its surface may be smooth or covered with projections that range from barely discernible to clearly visible. The basal socket is variable, being either simple or slightly depressed. This sensillum probably functions as a mechanoreceptor [4].

Sensilla trichodea II are also very common and are long, thick, and possess well-developed projections. A crescent-shaped pore is usually present on the anterior side of the base, and the tip is slightly narrowed and compressed, with the apical margin being either straight or curved. This type may also serve as a mechanoreceptor [4].

Sensilla chaetica are the largest sensilla on the antennae of *Cassida*. They are oriented perpendicularly to the surface of the antenna and are arranged in a sparse row along the apex of the distal antennomeres. They are long, conical, and unbranched, with slightly longitudinal ridges on their surface. Their bases are situated in circular, depressed, porous pits [4].

The most prominent ventral difference between the two species is the antennal groove located on the ventrolateral sides of the pronotum. Additionally, the prosternal process between the first pair of legs is broader in *C*. *palaestina* compared to *H*. *subferruginea* (Figures 7A–E and 8). At the region where the mesosternum connects with the prosternum, the distance between the legs is greater in *C. palaestina*, resulting in the second pair of legs being more widely spaced. In contrast, in *H. subferruginea*, the second pair of legs is positioned closer together at the junction of the mesosternum and prosternum Figure 7A–J).

**Figure 7:**
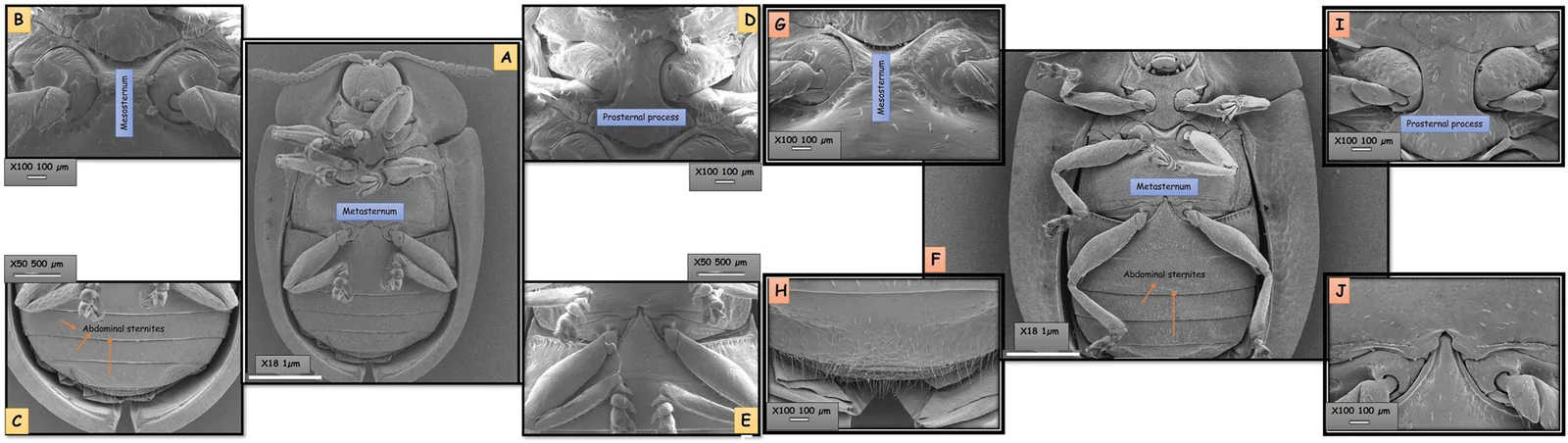
Comparative ventral morphology of *Hypocassida subferruginea* and *Cassida palaestina*. (A) Ventral morphology of *Hypocassida subferruginea,* general ventral view of *Hypocassida subferruginea*; (B) mesosternum of *Hypocassida subferruginea*; (C) abdominal sternites of *Hypocassida subferruginea*; (D) prosternal process of *Hypocassida subferruginea*; (E) metasternum and the first abdominal sternite of *Hypocassida subferruginea*; (F) ventral morphology of *Cassida palaestina,* general ventral view; (G) mesosternum of *Cassida palaestina*; (H) abdominal sternites of *Cassida palaestina*; (I) prosternal process of *Cassida palaestina*; (J) metasternum and the first abdominal sternite of *Cassida palaestina*.

**Figure 8:**
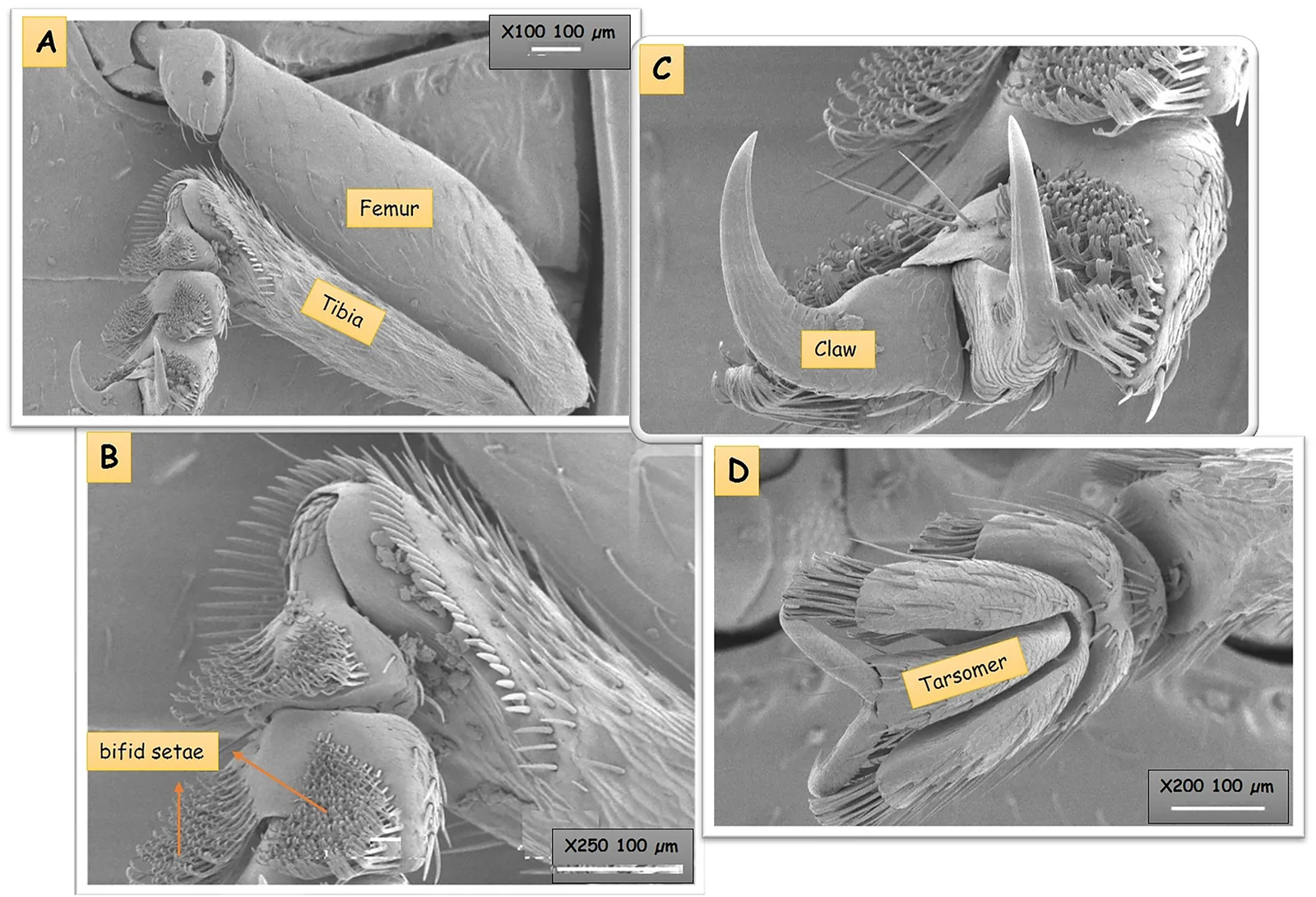
Morphology of the legs of *Hypocassida subferruginea*. (A) Entire hind leg; (B) apical notch and basal pad with bifid setae located beneath the leg; (C) single claw; (D) structure of the tarsomeres.

Regarding the leg morphology, the claws of both species are simple in structure, and the basal pad beneath the tarsus bears bifid setae. The tarsus is broader in *H. subferruginea* compared to *C*. *palaestina*. Moreover, in *H. subferruginea*, the fourth and the terminal tarsomeres bearing the claw are almost equal in length, whereas in *C. palaestina*, the fifth tarsomere bearing the claw is longer than the fourth tarsomere (Figures 8 and 9).

**Figure 9:**
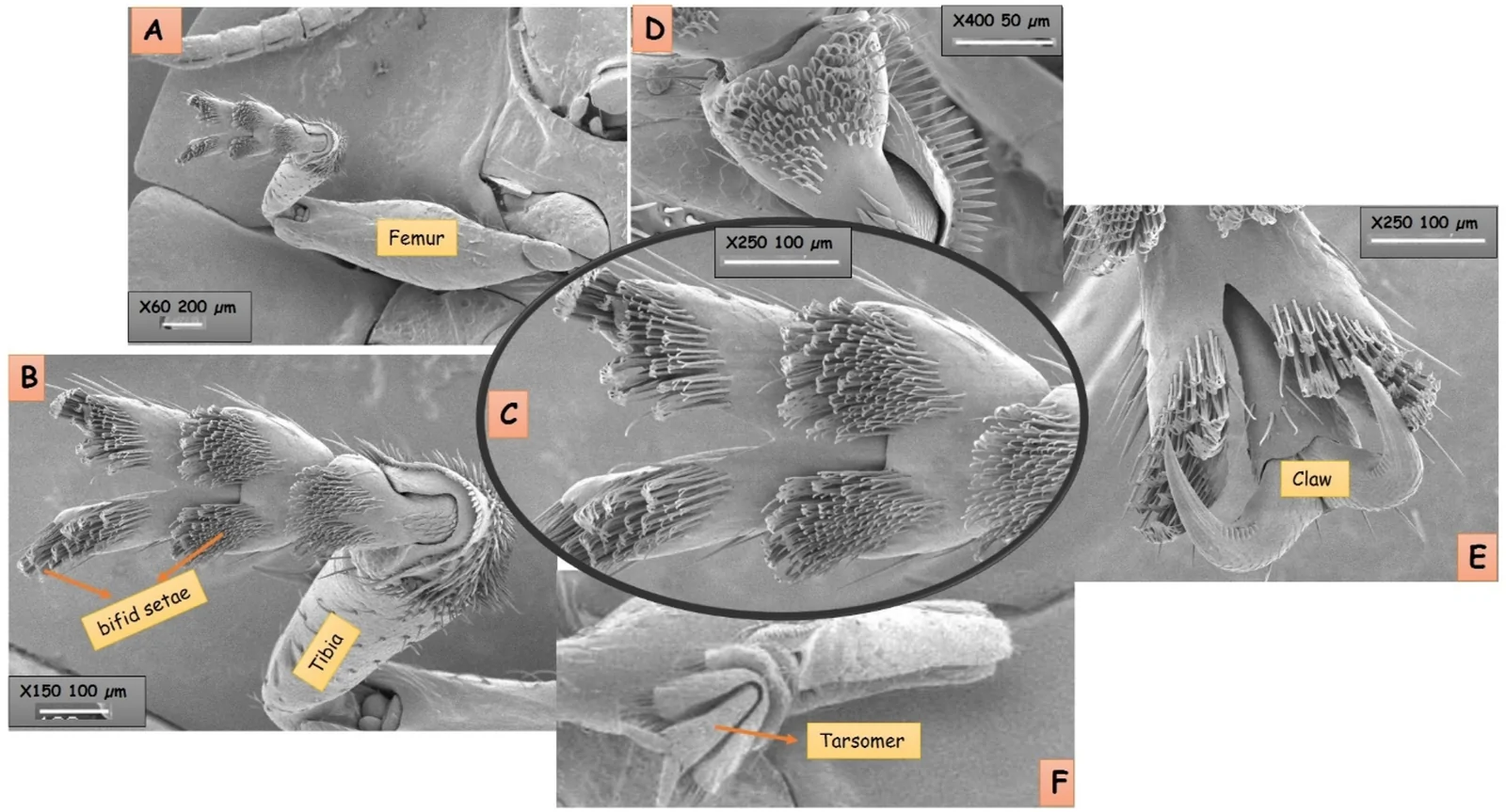
Morphology of the legs of *Cassida palaestina*. (A) Entire hind leg; (B, D) apical notch; (C) basal pad with bifid setae located beneath the leg; (E) single claw; (F) structure of the tarsomeres.

The comparative morphological analyses of *C*. *palaestina* and *H*. *subferruginea* reveal that subtle but consistent structural variations particularly in the pronotal configuration, antennal sensilla types, and tarsal proportions hold significant taxonomic and functional implications. The presence of a distinct antennal groove beneath the pronotum in *Hypocassida* constitutes a key diagnostic feature separating it from *Cassida*, highlighting a clear evolutionary divergence within the Cassidinae. Moreover, differences in sensilla distribution, density, and typology suggest potential adaptations to distinct ecological niches and sensory requirements, reflecting genus-specific behavioral and environmental interactions. The variations observed in the prosternal and mesosternal regions, together with leg and tarsal morphology, further support these distinctions and contribute valuable criteria for future phylogenetic and functional morphological studies within the Cassidini tribe. Overall, these findings emphasize the diagnostic and evolutionary significance of microstructural characters in delimiting closely related taxa and in understanding the adaptive diversification of leaf beetles.

The adult morphological distinctions between *C*. *palaestina* and *H*. *subferruginea* clearly demonstrate the taxonomic and functional divergence of these two genera. In order to obtain a more comprehensive understanding of their developmental morphology and to support these findings, the pupal stage of *H. subferruginea* and the larval stage of *C. palaestina* were examined in detail. While adult morphology provides valuable diagnostic characters, the examination of immature stages often reveals additional features critical for understanding phylogenetic relationships. Therefore, in the present study, the pupal morphology of *H*. *subferruginea* and the larval morphology of *C*. *palaestina* were investigated and compared.

Members of the genus *Hypocassida* are generally distributed in the Palaearctic region, yet their immature stages have been scarcely studied. The pupa can be described as follows: from a ventral view, numerous long cauliflower-shaped sensilla are prominent, especially on the prothorax, mesothorax, and legs, indicating incomplete development. On the thorax, the lateral scoli are triangular at the base and gradually taper toward thickened apices, bearing numerous spine-like projections (Figure 11). The shield-shaped structure at the posterior end of the body exhibits lateral projections that are relatively short and similar in length, whereas the posterior projections are approximately twice as long as the lateral ones. Around the head, the scoli are slender, branched, and considerably more numerous. From a dorsal perspective, the most striking features are the spiracular structures (Figures 10A–F, 12).

**Figure 10:**
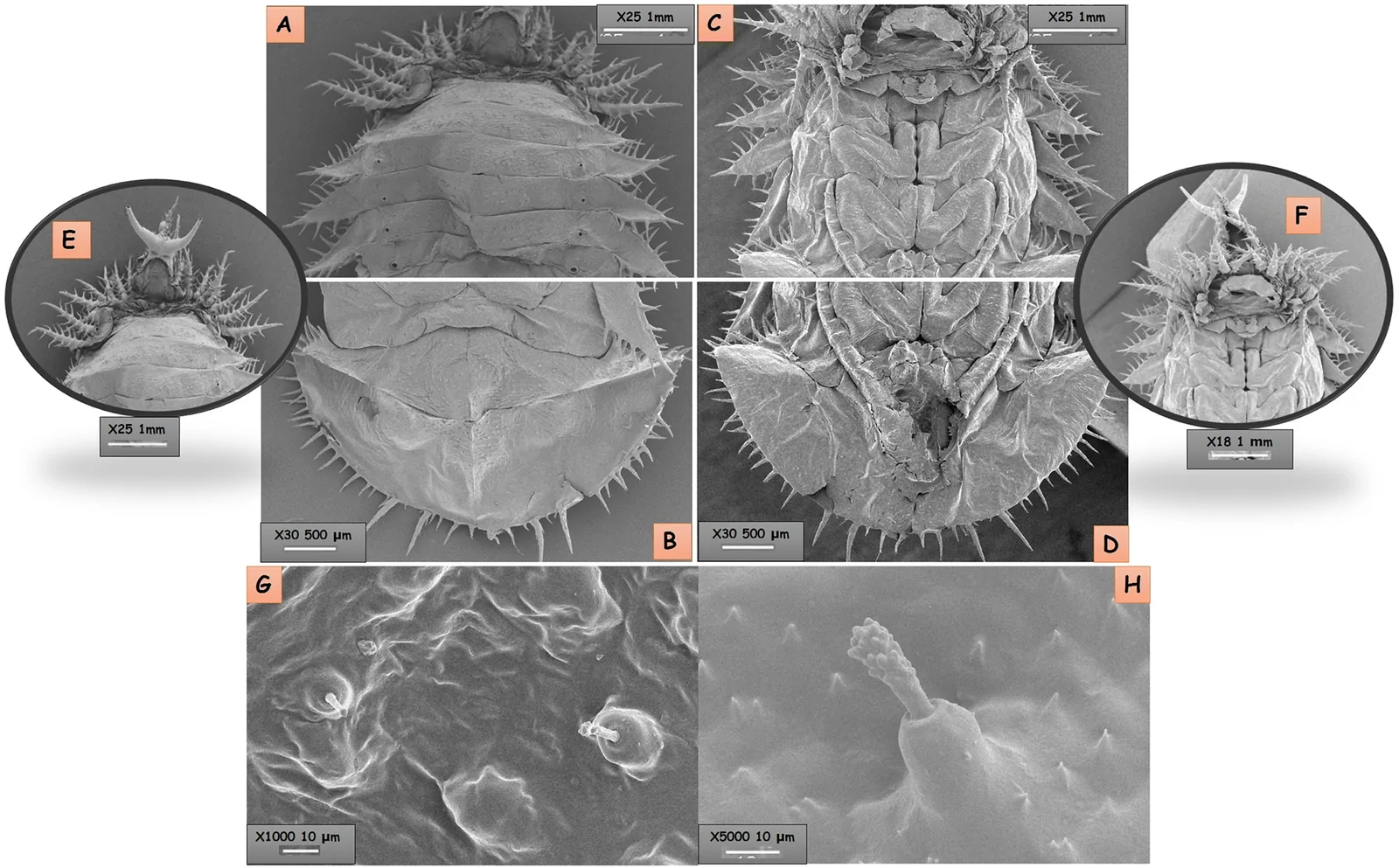
External morphology and sensilla of the *Hypocassida subferruginea* pupa. (A, B) Dorsal views of the *Hypocassida subferruginea* pupa; (C, D) ventral view of pupa; (E) dorsal view of the posterior region; and (F) ventral view of the anterior region, (G) Cauliflower-shaped sensilla on the legs of the ventral side of the *Hypocassida subferruginea* pupa, illustrating specialized sensory structures involved in substrate interaction, (H) Cauliflower-shaped sensilla surrounding the stigmas on the tergites, highlighting the arrangement of sensory organs associated with respiration and environmental sensing.

Different types of cauliflower-shaped sensilla are prominent on various regions of the pupa’s body. On the legs, the sensilla are slender and elongated, with lobes present only at the distal tips, whereas those surrounding the stigmas are shorter and exhibit more pronounced lobing at their apices (Figure 10G and H) (Figure 11).

**Figure 11:**
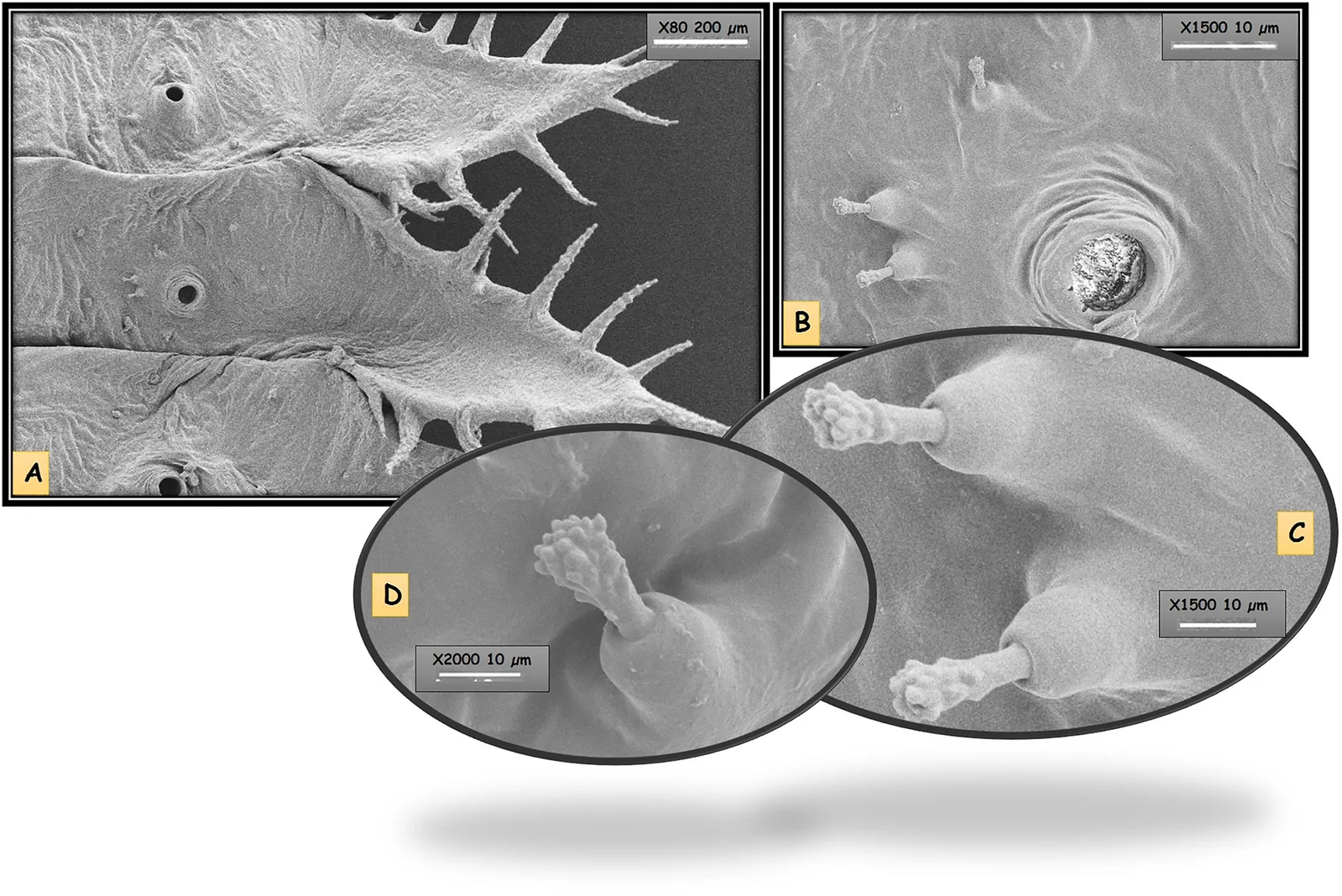
Abdominal tergites, stigmas, and sensilla of the *Hypocassida subferruginea* pupa. (A) Abdominal tergites I–III of *Hypocassida subferruginea* pupa, illustrating the arrangement of stigmal openings and lateral scoli; (B–D) detailed morphology of cauliflower-shaped sensilla surrounding the tergal stigmas, demonstrating characteristic sensory structures of the pupa.

The mouthparts of the pupa are well differentiated. The mandibles are deltoid in shape and bear several distinct teeth. The maxillary palps are relatively large, whereas the labial palps are slightly shorter, cylindrical, and terminate in a rounded shape. The ligula is broad and flat (Figure 12A–D).

**Figure 12:**
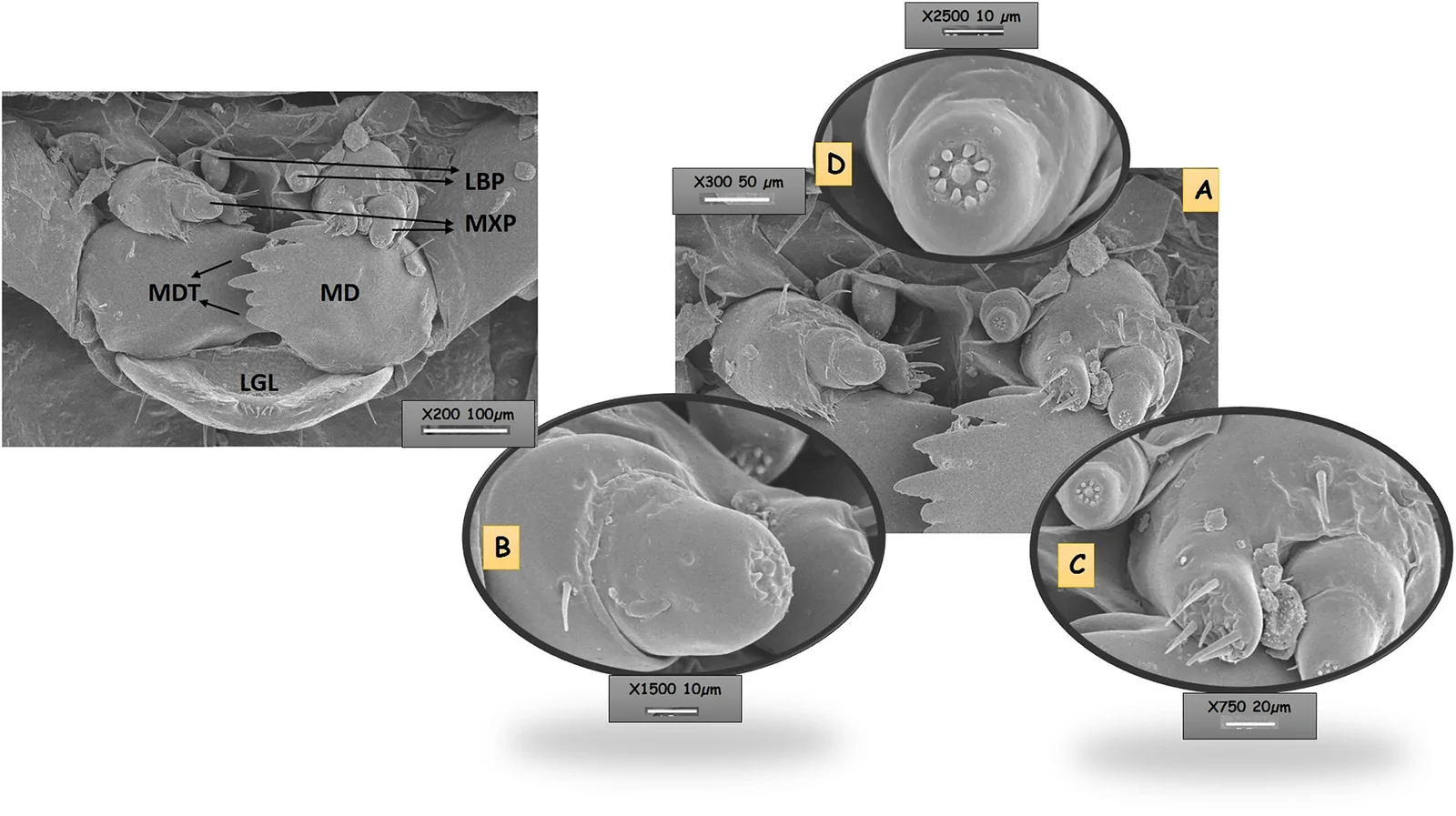
Mouthparts of the *Hypocassida subferruginea* pupa. (A) General structure of the mouthparts; (B, C) Maxillary palps; (D) Labial palps. (Abbreviations: MD, mandible; MDT, mandibular teeth; LGL, ligula; LBP, labial palps; MXP, maxillary palps).

The larva of *C*. *palaestina* was first described by Świętojańska et al. [21], providing a basis for species identification. The body is dorsoventrally flattened, oval in shape, and gradually tapers posteriorly, with the widest point located between the meso- and metanotum, corresponding to the mid-body region. The larva possesses 16 pairs of lateral scoli and a single pair of long supra-anal processes approximately equal in length to the body [21].

Our observations reveal considerable variation in the lengths of the lateral scoli: the most anterior and posterior scoli are the longest, whereas those in the mid-body region are relatively uniform, showing minor individual differences. The first pair of lateral scoli bears two simple lateral branches, while the remaining scoli are unbranched but carry several elongated, cauliflower-shaped sensilla. These sensilla are notable not only for their length and lobed apices but also for their arrangement and distribution along the body; in some scoli, they are densely aligned in a single row, whereas in others, they are more sparsely and irregularly distributed. Particularly, the posterior abdominal scoli bear shorter sensilla with prominent apical lobes, while anterior scoli possess longer sensilla with slender, finer lobes (Figure 13A–E).

**Figure 13:**
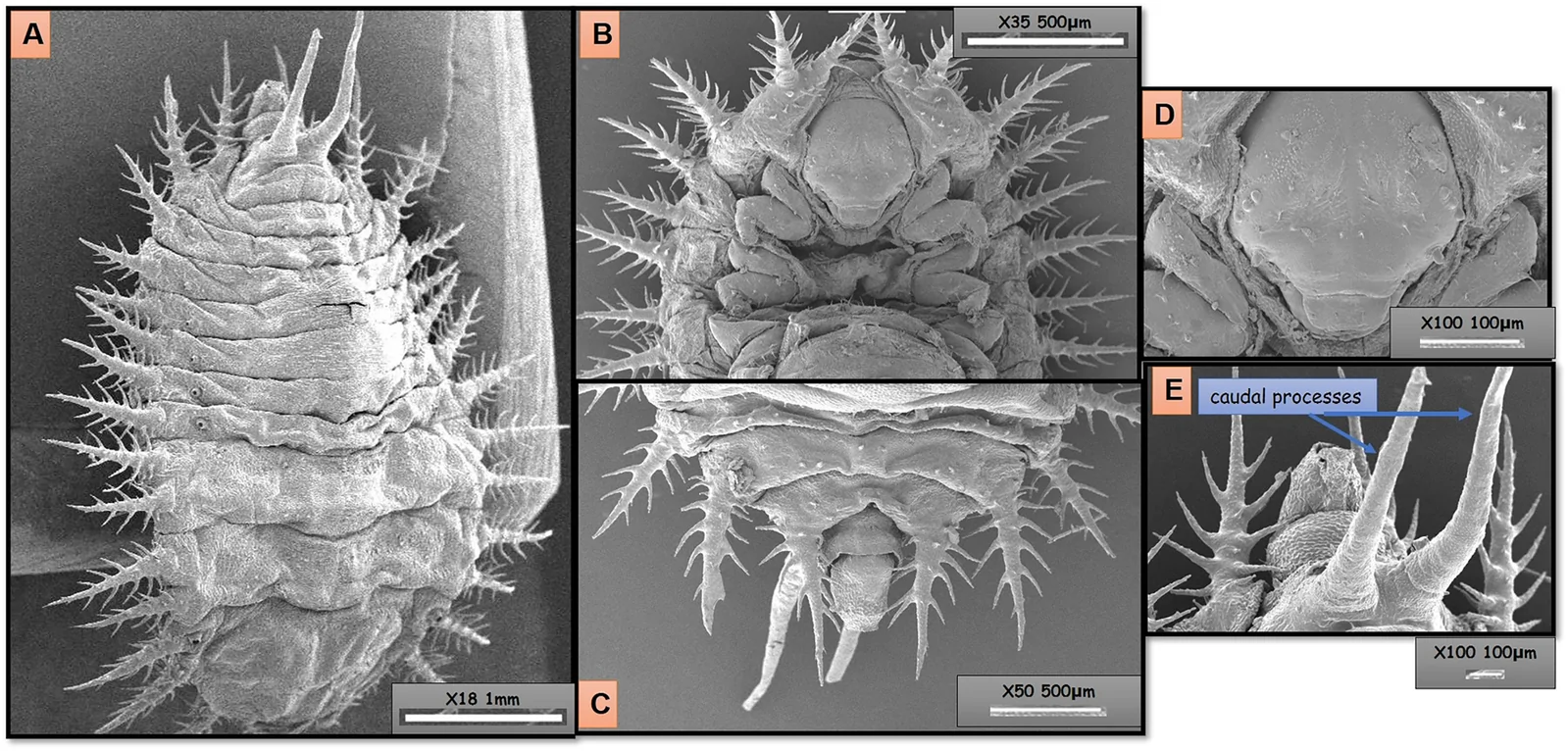
General morphology of the *Cassida palaestina* larva. (A) General body shape of the *Cassida palaestina* larva; (B) ventral view of the head region; (C) ventral view of the posterior region showing the anal opening; (D) dorsal view of the concealed head region; E. Structure of the paired caudal processes.

These morphological details have not been reported in previous studies and provide novel insights into the distinguishing characteristics of the larva, as well as its potential sensory mechanisms for environmental perception.

In the larva, the head structures, including the labrum, stemma, antennae, and caudal processes, are well-defined and prominent (Figure 13A–E).

The comparative examination of adult, pupal, and larval stages of *C*. *palaestina* and *H*. *subferruginea* underscores the taxonomic and functional divergence within the Cassidinae. Subtle yet consistent structural differences in the pronotum, antennal sensilla, tarsal proportions, and prosternal-mesosternal regions highlight genus-specific adaptations and evolutionary separation. Detailed analysis of immature stages further reveals previously unreported features, including the distribution, density, and morphology of cauliflower-shaped sensilla on the pupal body of *H. subferruginea* and the lateral scoli and supra-anal processes of *C. palaestina* larvae (Figures 14 and 15). These microstructural characters such as the branched anterior lateral scoli, variation in sensilla length and apical lobation, and well-defined head structures including labrum, stemma, antennae, and caudal processes provide novel diagnostic traits and suggest functional adaptations for sensory perception and environmental interaction. Collectively, these findings emphasize the importance of integrating multiple developmental stages to fully capture the morphological diversity, ecological specialization, and evolutionary trajectories of leaf beetles, offering valuable criteria for future taxonomic, phylogenetic, and functional studies within the Cassidini tribe.

**Figure 14:**
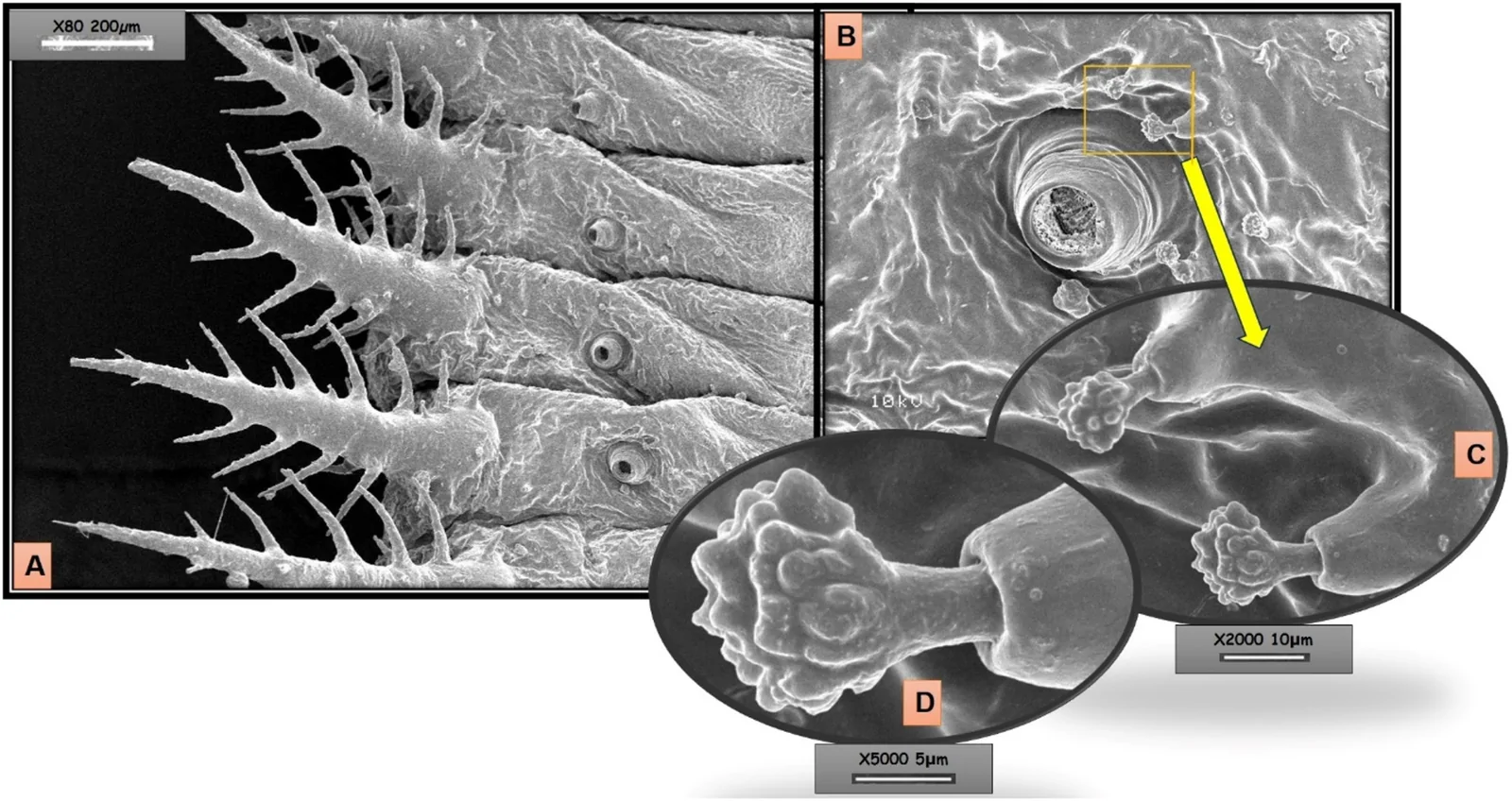
Abdominal sensilla of the *Cassida palaestina* larva. Cauliflower-shaped sensilla located near the stigma on the abdominal sternites of the *Cassida palaestina* larva.

**Figure 15:**
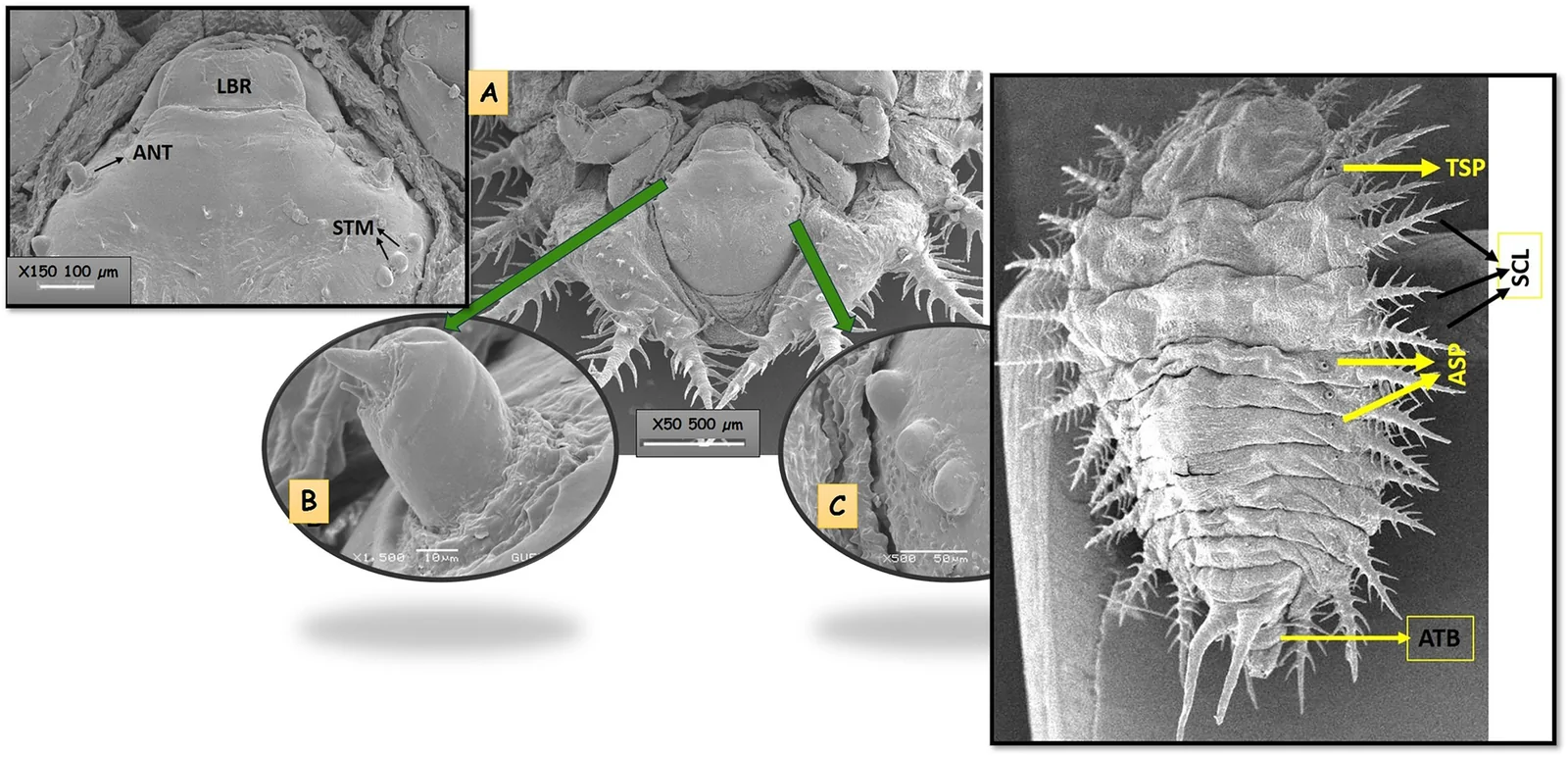
Head morphology and sensory structures of the *Cassida palaestina* larva. (A) General view of the head of the *Cassida palaestina* larva; (B) antenna; (C) stemma (abbreviations: TSP, thoracic spiracle; SCL, scolus; ASP, abdominal spiracles; ATB, anal tube; ANT, antenna; LBR, labrum; STM, stemma).

### 3.1 Habitat suitability under current climatic conditions

The discriminatory performance of the MaxEnt models was evaluated using the area under the receiver operating characteristic curve (AUC). Mean AUC values were 0.942 for *H*. *subferruginea* and 0.997 for *C*. *palaestina* (Figure 16C and D). Because both values exceeded 0.90, the models demonstrated excellent ability to distinguish suitable from unsuitable habitats under current climatic conditions. In particular, the AUC value of the *C*. *palaestina* model, which approached 1.0, indicated exceptionally high predictive performance.

**Figure 16:**
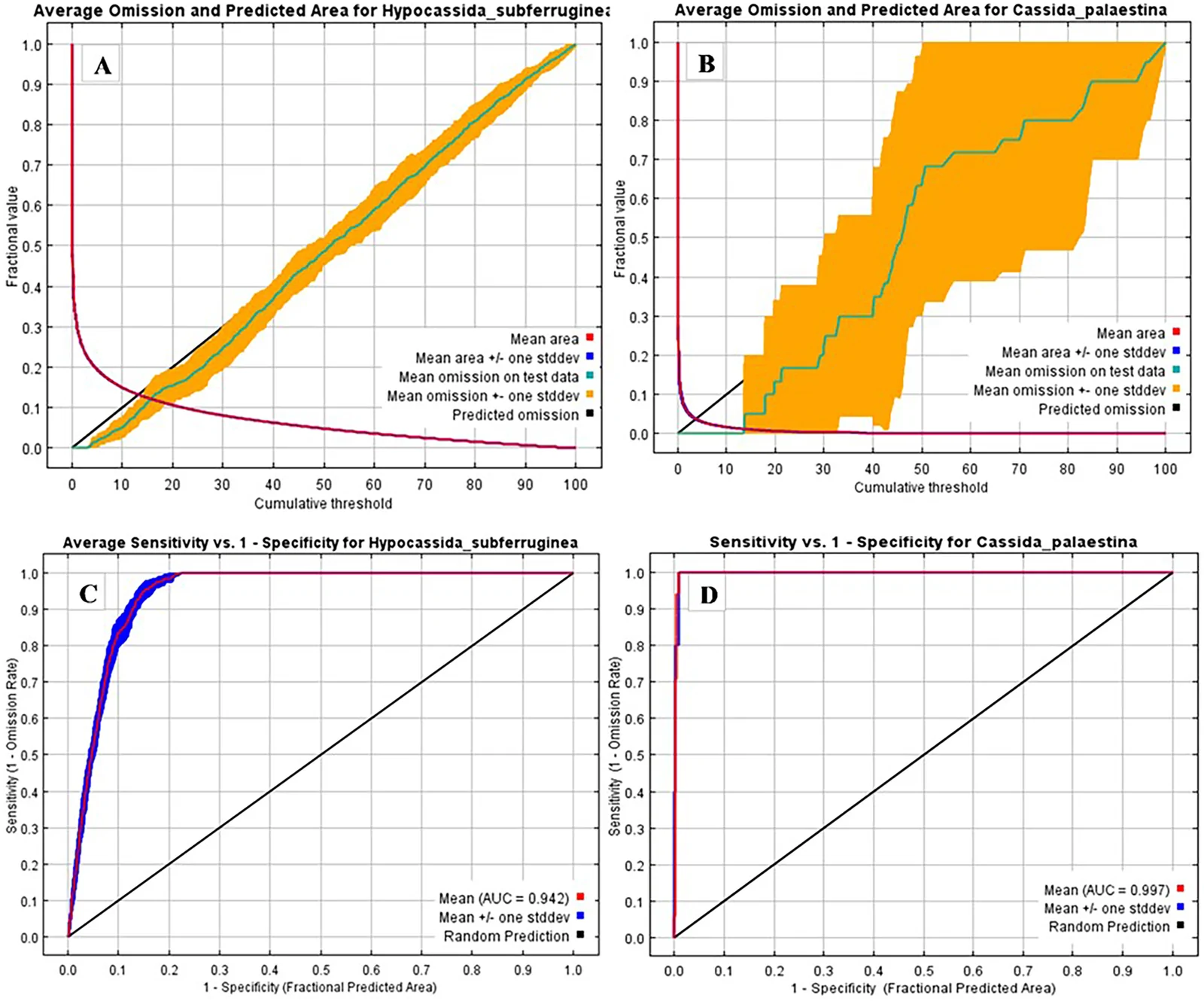
Evaluation of the accuracy of MaxEnt models in predicting the potential geographic distributions of *H. subferruginea* and *C. palaestina*. Model evaluations, (A–C) average omission and predicted area: *H. subferruginea*, *C. palaestina*; ROC curve (D–F): *H. subferruginea*, *C. palaestina*.

The average omission and predicted area plots showed that the observed omission rate for *H*. *subferruginea* generally remained close to the expected omission rate (Figure 16A), supporting adequate model calibration. For *C. palaestina*, the wider variability across threshold values may have resulted from the distribution of occurrence records or differences among cross-validation subsets (Figure 16B). Nevertheless, the ROC curves were clearly positioned above the random-prediction line, confirming that both models had strong predictive ability compared with random classification.

The relative importance of the bioclimatic variables was evaluated using jackknife analysis. Minimum Temperature of the Coldest Month (bio6), Isothermality (bio3), and Annual Mean Temperature (bio1) were the most influential variables in the *H. subferruginea* model. In contrast, Mean Temperature of the Wettest Quarter (bio8), Mean Temperature of the Driest Quarter (bio9), and Precipitation of the Driest Quarter (bio17) were more important in the *C. palaestina* model (Figure 17). These findings indicate that the distribution of *H. subferruginea* is governed primarily by the thermal regime, whereas the distribution of *C. palaestina* is shaped by the combined effects of temperature and precipitation.

**Figure 17:**
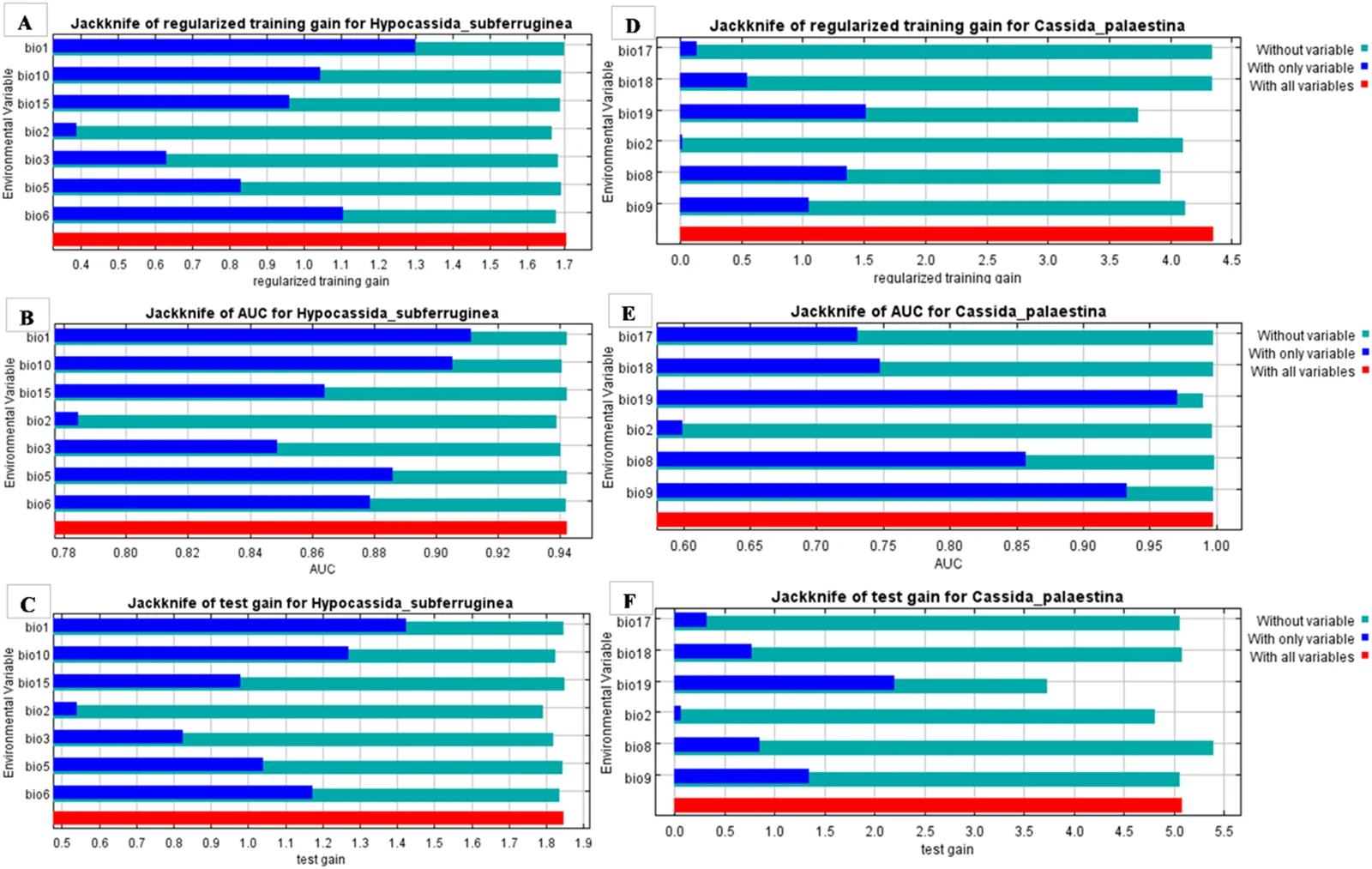
Jackknife of regularized training gain: A and D for *H. subferruginea* and *C. palaestina*; AUC: B and E for *H. subferruginea* and *C. palaestina*; test gain: C and F for *H. subferruginea* and *C. palaestina*.

The jackknife results revealed both the information provided by each variable when used independently and the information lost when that variable was omitted from the model (Figure 17). The overall consistency among regularized training gain, AUC, and test gain indicated that the selected variables had stable explanatory power. Nevertheless, variable contributions should not be interpreted solely as independent effects; interactions and potential correlations among the bioclimatic variables should also be considered.

The response curves of the environmental variables revealed clear differences between the climatic requirements of the two species (Figure 18). For *H*. *subferruginea*, bio10 exhibited a plateau-shaped response with high suitability at intermediate values, habitat suitability increased with increasing values of bio6 and bio5, and bio1 showed a unimodal relationship. In contrast, habitat suitability for *C*. *palaestina* decreased with increasing bio17 and bio2, increased with increasing bio9, and reached its maximum within a specific temperature range for bio8. These contrasting responses support the existence of different ecophysiological tolerance ranges and indicate that the potential distributions of the two species are governed by different climatic constraints.

**Figure 18:**
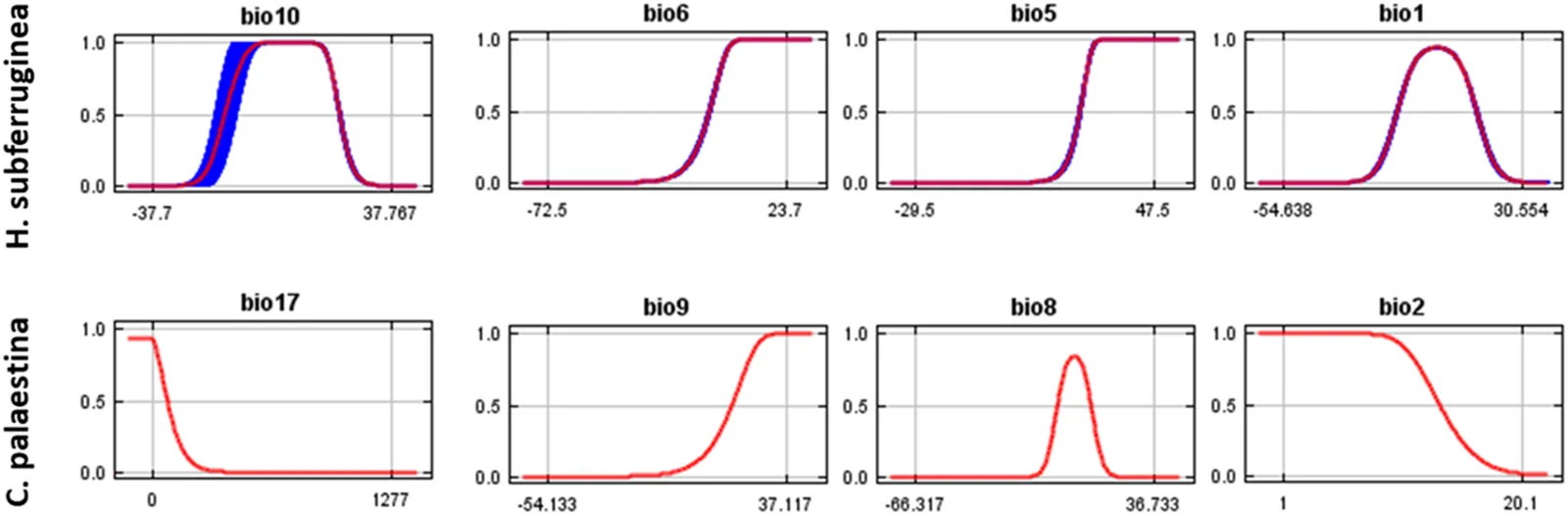
Response curves of key environmental variables in the MaxEnt model for *H*. *subferruginea* and *C*. *palaestina*.

Current Global and National (Türkiye) Distributions of *H*. *subferruginea* and *C*. *palaestina* Based on MaxEnt modeling using global occurrence records of the species, it was determined that under current climatic conditions approximately 24 % of Türkiye provides the most suitable ecological conditions for the distribution of *H. subferruginea*. The map presented in Figure 16B classifies habitat suitability into four standard categories according to model outputs: highly suitable, suitable, moderately suitable, and unsuitable areas. These categories represent the potential habitat suitability of *H. subferruginea* in Türkiye under present climatic conditions. The color scale used on the map reflects different levels of habitat suitability for the species.

Areas classified as highly suitable (red) are mainly concentrated in the northern belt of Türkiye, particularly along the Black Sea coastal region, the northern parts of the Marmara Region, and certain areas of Eastern Anatolia (especially around Erzurum, Erzincan, Bingöl, and Van). The moderate temperatures and relatively high humidity prevailing in these regions indicate that the species prefers humid and temperate climatic conditions. Suitable (orange) and moderately suitable (light green) habitats are distributed across the inner parts of Western Anatolia, the northern and eastern sections of Central Anatolia, and transitional zones of Eastern Anatolia. These areas represent habitats where the species may occur but where conditions are not optimal. In such regions, the presence of the species may vary depending on the suitability of local microclimatic conditions.

In contrast, unsuitable areas (white) are predominantly located in the semi-arid regions of Central and Southeastern Anatolia, the Tuz Lake Basin, the Mediterranean coastal belt, and along high-altitude mountainous areas. In these regions, extreme temperature values and low humidity are considered the main limiting factors restricting species establishment. Overall, the model reveals that *H. subferruginea* favors temperate and moderately humid climates, with its most suitable habitats concentrated in the northern and northeastern parts of Türkiye. This distribution pattern is consistent with the ecological requirements of the species. Indeed, Bardin and Timraleev [35] reported that the species is widely distributed in mesophilic (moderately moist) and heliophilic (sun-loving) habitats, and that it can also occur in dry grasslands, agricultural areas, valley slopes, steppe, and steppe-like habitats [35].

The model results obtained for *C*. *palaestina* indicate that under current climatic conditions, approximately 21 % of Türkiye constitutes the most suitable habitat for the species, with these areas being predominantly concentrated in the southern belt of the country (Figure 16D). According to the color scale used on the map, highly suitable areas (red) are mainly distributed across the Mediterranean and Southeastern Anatolia regions. In particular, the provinces of Antalya, Mersin, Adana, Hatay, Osmaniye, Kahramanmaraş, Gaziantep, Şanlıurfa, Mardin, Batman, and Siirt represent the most suitable habitats for the species. These regions share common climatic characteristics, including high mean temperatures, mild winter conditions, and arid to semi-humid climates. This pattern supports the notion that *C. palaestina* is adapted to warm and relatively dry climatic conditions [36].

Suitable (orange) and moderately suitable (light green) areas occur in the coastal and inland transitional zones of the Aegean Region (particularly around İzmir, Manisa, Aydın, and Muğla), along the southern margins of Central Anatolia (Konya, Karaman, Niğde), and in the lower altitudinal belts of Eastern Anatolia. In these areas, the occurrence of the species is closely associated not only with climatic conditions but also with the availability of host plants. In contrast, unsuitable areas (white) are mainly distributed throughout the Black Sea Region, the northern parts of Central Anatolia, the high-altitude areas of Eastern Anatolia, and around the Tuz Lake Basin. In these regions, low temperatures and high humidity levels are considered the primary limiting factors restricting the distribution of the species. Overall, the model demonstrates that *C. palaestina* favors warm, semi-arid, and mild climatic conditions, and that the most suitable habitats are concentrated in the southern regions of Türkiye, particularly within the Mediterranean and Southeastern Anatolia regions.

The distribution predictions generated using the MaxEnt model indicate that *H*. *subferruginea* and *C. palaestina* exhibit distinct ecological responses to climatic variables in terms of habitat suitability across Türkiye (Figure 19A–D). The distribution patterns of the two species clearly reflect different geographical and ecological preferences under current climatic conditions. A comparison of the habitat suitability maps reveals a pronounced north–south ecological gradient within Türkiye.

**Figure 19:**
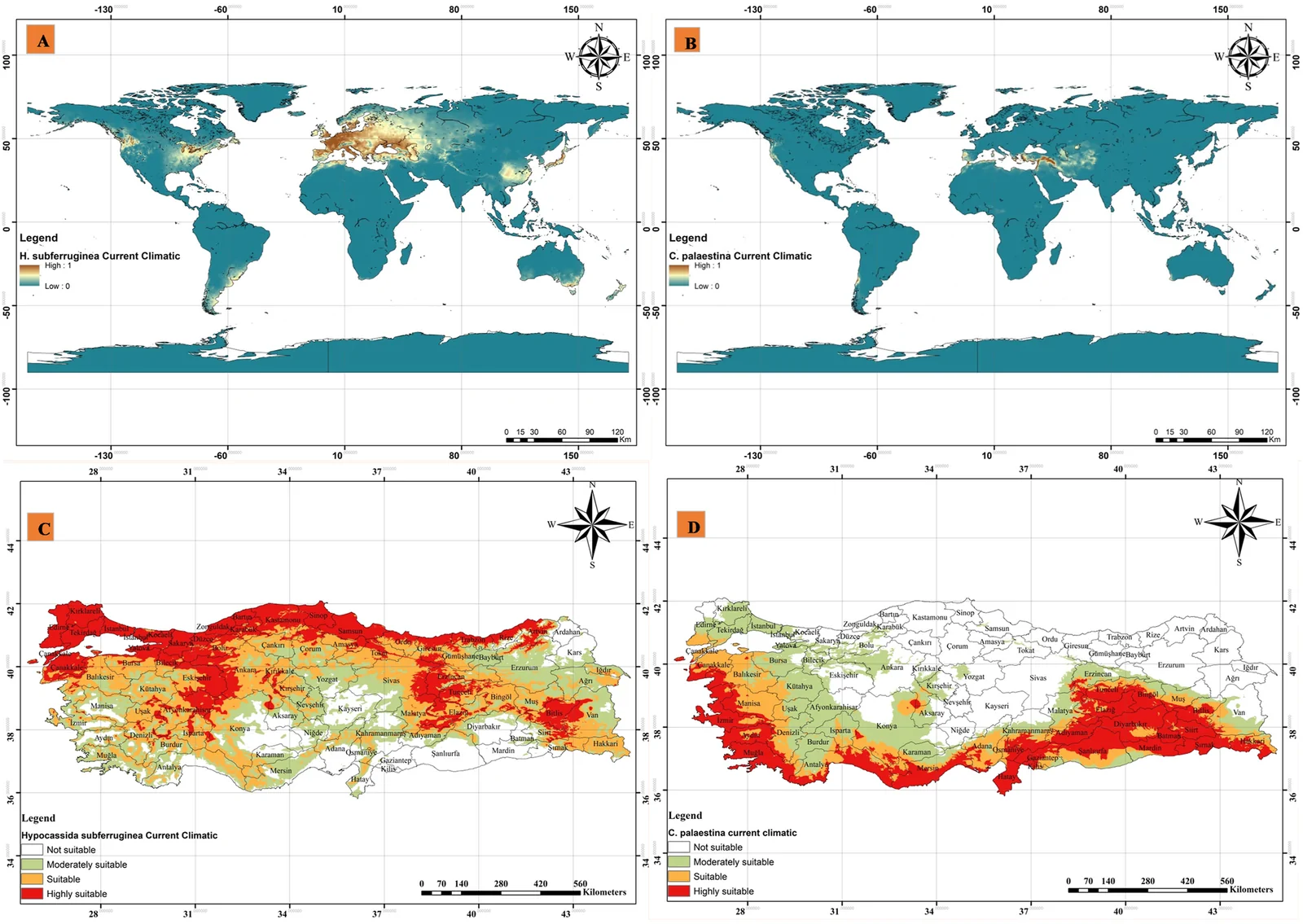
Predicted habitat suitability and potential geographic distributions of *Hypocassida subferruginea* and *Cassida palaestina* under current climatic conditions. (A) Suitable areas for the distribution of *H*. *subferruginea* under current climatic conditions at the global scale; (B) muitable areas for the distribution of *C*. *palaestina* under current climatic conditions at the global scale; (C) map showing the predicted habitat suitability levels for *H*. *subferruginea* across Türkiye under current climatic conditions. On the map, not suitable indicates unsuitable areas, moderately suitable indicates partially suitable areas, suitable indicates suitable areas, and highly suitable indicates the most suitable habitats; (D) map showing the predicted habitat suitability levels for *C*. *palaestina* across Türkiye under current climatic conditions. On the map, not suitable represents unsuitable areas, moderately suitable represents partially suitable areas, suitable represents suitable areas, and highly suitable represents the most suitable habitats.

Habitat suitability for *H. subferruginea* reaches its maximum in cooler and more humid regions, whereas suitability for *C. palaestina* peaks in warmer and drier areas. This contrasting distribution pattern suggests that the two species possess different ecophysiological tolerance ranges. Furthermore, the occurrence of suitable habitats for *H. subferruginea* in higher-altitude regions indicates a greater tolerance to cold stress, while the concentration of *C. palaestina* in low-altitude, coastal, and plain areas reflects an adaptation to heat stress. Consequently, the potential geographical overlap between the two species appears to be limited, indicating a biogeographically complementary distribution pattern. Overall, the model results demonstrate that *H. subferruginea* favors humid and temperate climatic conditions, whereas *C. palaestina* prefers warm and semi-arid climates. This ecological differentiation suggests that the two species may be effective in different geographical regions in terms of biological control potential. Therefore, considering the climate-based ecological suitability of these species is crucial for the planning and implementation of future biological control programs.

The present study demonstrates that integrating microstructural morphology (SEM) with climate-based habitat suitability modeling (MaxEnt) provides a powerful framework for understanding both taxonomic separation and ecological divergence within Cassidini. Adult SEM characters especially the pronotal antennal grooves diagnostic for *Hypocassida* together with consistent differences in sensilla patterns, sternal morphology, and tarsal proportions, clearly support morphological and functional differentiation between *H. subferruginea* and *C. palaestina*.

A major contribution of this work is the first description of the pupal stage of *H. subferruginea*, complemented by detailed SEM documentation of immature-stage microcharacters (e.g., cauliflower-shaped sensilla, scoli configuration, and spiracular-associated structures). These characters expand the diagnostic toolkit available for Cassidini and emphasize the importance of immature stages for resolving relationships and evolutionary patterns within Cassidinae.

MaxEnt outputs further indicate that the two species respond differently to climatic constraints and exhibit a marked biogeographic partitioning across Türkiye. Under current conditions, *H. subferruginea* is predicted to attain maximum suitability in cooler and more humid northern and northeastern regions, while *C. palaestina* is concentrated in warmer and drier southern belts, implying limited potential overlap and a complementary distribution pattern. Variable contribution analyses suggest that temperature is the dominant driver for *H. subferruginea*, whereas *C. palaestina* is shaped by both temperature and precipitation, consistent with their contrasting ecological affinities. The microstructural characters documented in the present study extend beyond simple species descriptions and contribute to the systematic framework of Cassidinae. In particular, the presence of deep pronotal antennal grooves in *Hypocassida*, together with differences in antennal sensilla, sternal morphology, tarsal proportions, and immature-stage structures, provides additional diagnostic characters supporting the separation of *Hypocassida* from *Cassida*. These characters complement the traditional external morphological features used in Cassidinae taxonomy and may represent informative morphological markers for future phylogenetic studies integrating both morphological and molecular datasets.

Collectively, these results underscore that microdiagnostic morphological traits and macroecological niche patterns converge to reveal genus- and species-specific adaptations. Considering these climate-based suitability patterns is expected to be particularly valuable for future monitoring, conservation planning, and regionally targeted biological control strategies, where accurate identification and ecological suitability are critical. Although the present study provides valuable information on climatic habitat suitability, it does not evaluate ecological interactions or biological control efficiency. Therefore, the potential applications suggested here should be considered preliminary and require validation through future ecological and experimental studies.
